# Sustainable Nonwoven Scaffolds Engineered with Recycled Carbon Fiber for Enhanced Biocompatibility and Cell Interaction: From Waste to Health

**DOI:** 10.1021/acsabm.4c01475

**Published:** 2025-02-17

**Authors:** Jonas Naumann, Kresten Singer, Siddharth Shukla, Alok Maurya, Stefan Schlichter, Imre Szenti, Akos Kukovecz, Amit Rawal, Mareike Zink

**Affiliations:** † Research Group Biotechnology and Biomedicine, Peter-Debye-Institute for Soft Matter Physics, Leipzig University, Linnéstraße 5, 04103 Leipzig, Germany; ‡ Department of Textile and Fibre Engineering, Indian Institute of Technology Delhi, Hauz Khas, New Delhi 110016, India; § Faculty of Mechanical and Process Engineering, Makers labs Recycling & AI, Technische Hochschule Augsburg, University of Applied Sciences, An der Hochschule 1, 86161 Augsburg, Germany; ∥ Interdisciplinary Excellence Centre, Department of Applied and Environmental Chemistry, University of Szeged, Rerrich Béla tér 1., 6720 Szeged, Hungary

**Keywords:** nonwovens, recycled carbon fibers, polypropylene, biomaterials, bronchial epithelial cells, artificial lung tissue scaffolds

## Abstract

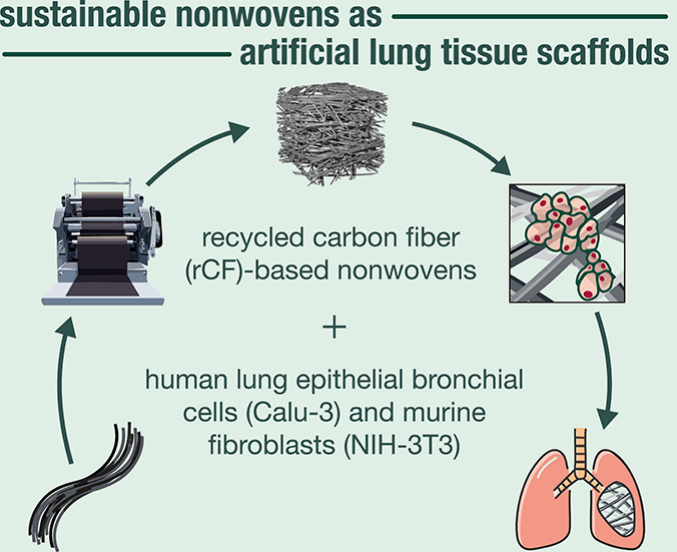

Carbon fibers, driven by ever-increasing demand, are contributing to a continuous rise in the generation of waste and byproducts destined for landfills or incineration. Recycling carbon fibers presents a promising strategy for reducing carbon emissions and conserving resources, thus contributing to more sustainable waste management practices. Discovering applications of recycled carbon fibers (rCFs) would inevitably accelerate the targeted integration of sustainable materials, fostering a circular economy. Herein, we have engineered rCF-based needlepunched nonwoven scaffolds and their blends with polypropylene (PP) fibers, providing the first example of investigating their interactions with human lung epithelial cells (Calu-3) and murine fibroblast cells (NIH/3T3). To promote the adsorption of extracellular matrix proteins such as laminin, these three-dimensional (3D) nonwoven scaffolds are designed and developed to feature tunable porous characteristics and wetting properties. Although cell adhesion and laminin adsorption are minimal on PP fibers, cells are preferentially organized on the rCFs. These nonwovens, composed exclusively of rCFs or their blends with PP fibers, exhibit no cytotoxic effects, with both cell types showing proliferation on the scaffolds and a progressive increase in cell numbers over time. Cell viability and apoptosis assays are also employed to comprehensively evaluate biocompatibility. Thus, our study proves rCF-based nonwoven scaffolds as potential candidates for artificial lung tissue scaffolds.

## 1 Introduction

Tissue engineering aims to revive damaged tissues and organs by integrating biological knowledge with engineering principles, utilizing basic tools such as cells, scaffolds, and growth factors.^1^ The engineering design of a three-dimensional (3D) porous scaffold provides a suitable environment for modulating cell attachment, proliferation, and differentiation.^2,3^ Further, these 3D porous scaffolds can dictate the structure–property relationship of the newly formed tissue.^4^ Specifically, the porous nature of 3D scaffolds profoundly impacts cell spatial organization, cellular morphology, and tissue regeneration.^2^ For instance, scaffolds with higher porosities demonstrate significant efficacy in cartilage regeneration compared to those with lower porosities.^5^

Replacing organs and tissues is crucial, as their demand far exceeds the supply.^6^ Given that lung and heart diseases are leading causes of death worldwide,^7^ the ability to replace affected tissues could revolutionize treatment in this field. A potential approach to address this challenge involves decellularized biological scaffolds crafted from porous extracellular matrix (ECM) material.^8,9^ Perhaps a key application would be the replacement of pulmonary tissue for patients suffering from severe lung diseases such as chronic obstructive pulmonary disease (COPD), pneumoconiosis, pulmonary fibrosis, or lung cancer.^10,11^ However, there are still many challenges and limitations to overcome before decellularized scaffolds can be used for human lung transplantation. Some of these challenges include the immaturity of the vascular bed and the epithelial cells, the risk of infection and inflammation, the ethical and regulatory issues, and the scalability and availability of donor lungs.^12,13^ In addition, the decellularization process can modify the mechanical properties of the ECM, which in turn can impact the mechanical properties of the implant.^12^

Artificial or synthetic scaffolds provide an alternative approach, with the advantage of tailoring biological, structural, and mechanical properties to match the target tissue.^11,14^ Current techniques, including 3D bioprinting, cryogelation, and solvent-casting, have yet to overcome challenges, such as reliably reproducing complex tissue structures while ensuring good biocompatibility of the underlying biomaterials.^14,15^ For example, despite the increasing medical need for tracheal replacement,^16^ reconstructive strategies for the trachea remain challenging due to its complex structure, which requires a unique combination of lateral rigidity and longitudinal flexibility.^17^ An important biomaterial feature besides biocompatibility is the ability to promote cell growth and a large surface area for adequate cell adhesion.^18^ Within this context, fiber networks, in the form of needlepunched nonwoven materials, serve as ubiquitous scaffolds, offering mechanical stability and 3D porous geometry, which enable the modulation of cell attachment, proliferation, and differentiation.^19,20^

Needlepunched nonwovens form a unique class of 3D interconnected porous scaffolds created by the action of barbed needles that reorientate a portion of fibers from the surface to the thickness direction.^21^ These materials, made from recycled carbon fibers, have facilitated second-life opportunities by enabling various structural and nonstructural-based applications.^22^ However, the growing body of literature on recycled carbon fiber (rCF)-based needlepunched nonwoven materials has not endeavored to engineer them as scaffolds for cell culture. A combinatorial strategy involving the 3D features of needlepunched nonwoven materials^23^ and biocompatible carbon fibers facilitates cell attachment to the scaffold surface, promoting cell growth and maintaining differentiated cell phenotypes.^24^

The central aim of this study is to investigate the interaction of needlepunched nonwoven scaffolds composed exclusively of rCFs and their blends with polypropylene (PP) fibers, using various cell types, including human lung epithelial cells (Calu-3) and murine fibroblast cells (NIH/3T3). To the authors’ best knowledge, this is the first attempt to utilize rCF-based needlepunched nonwoven materials as scaffolds in tissue engineering. In addition extensive material characterization crucial for their application as biomaterials, our findings demonstrate that cells effectively adhere, spread, and proliferate across scaffolds with diverse morphological characteristics, which is essential for cellular colonization. Given that cell adhesion is facilitated by the adsorption of extracellular matrix (ECM) proteins, which is crucial for biomaterial integration within the human body, our study establishes the groundwork for utilizing recycled nonwoven materials in medical and biomedical engineering applications. Further, the combination of hydrophilic carbon fibers and hydrophobic PP fibers could offer a balanced scaffold such that the hydrophilic carbon fibers provide sites for initial cell attachment while the large PP fibers contribute to mechanical stability and enhance the porous structure essential for cell growth.^5^

## 2 Materials and Methods

### 2.1 Preparation of Nonwoven Samples

**Figure 1:**
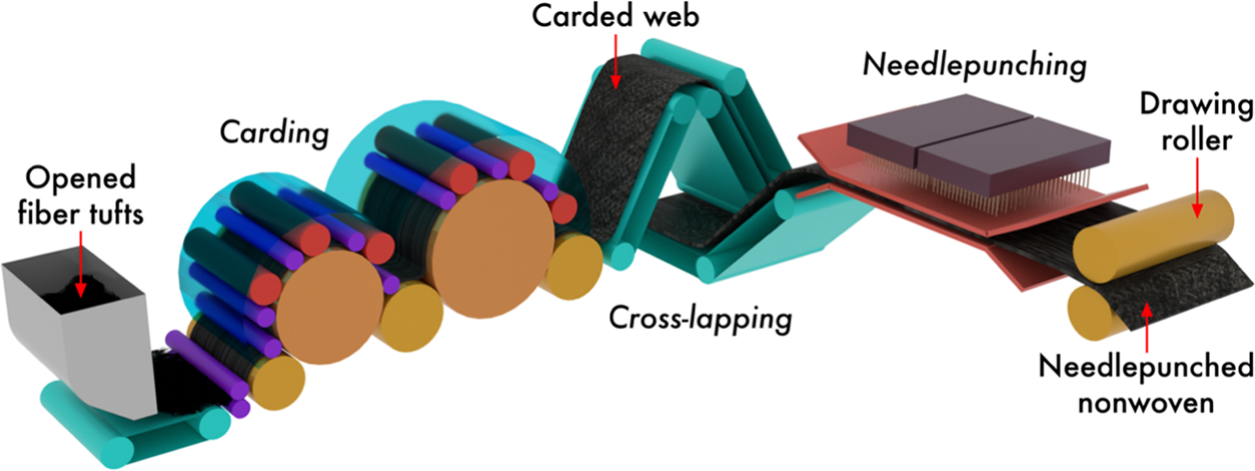
Schematic representing the preparation of recycled carbon fiber-based needlepunched nonwoven materials.

Three sets of needlepunched nonwoven samples, A, B, and C, were fabricated by blending fine recycled carbon fibers (rCFs) sourced from various types of waste, including composites from diverse manufacturing processes and end-of-life products and coarse polypropylene (PP) fibers in varying proportions. Here, sample A consists exclusively of rCF, sample B consists of 50% rCF and 50% PP fibers, and sample C comprises 10% rCF blended with 90% PP fibers. Initially, the fibers were proportionally weighed to create the desired blend ratio and then processed through a bale opener, followed by a carding willow and a double fine opener. The opened fiber tufts were then fed into the carding machine using a combination of chute feed and vibration feed system, along with a weighing belt to ensure uniform feed. Subsequently, the fibers were processed into a web using a compact roller card and directly linked cross-lapper (see Figure 1). The resultant web was then passed through two sets of needle boards on a custom-built needlepunching machine, maintaining a punch density of 60 punches/cm^2^ and a needle penetration depth of 8 mm, forming a preneedled nonwoven structure. The final samples were analyzed for their 3D morphological and wetting characteristics. Table S1 shows the physical properties of the prepared nonwoven samples.

### 2.2 X-ray Micro-Computed Tomography (microCT) Analysis

The 3D structural parameters of the nonwoven samples were determined through X-ray microCT analysis. Initially, rectangular specimens were mounted on the sample stage of the Skyscan 2211 (Bruker, Belgium) X-ray microCT system. A tungsten target with a source voltage of 50 kV and a source current of 600 μA was then used to focus the X-rays on the sample stage. Subsequently, the stage was rotated to 194° with an angular step of 0.1°, and a suitable exposure time was maintained to capture the projections. These projections were reconstructed to obtain data sets of 2300–2400 images with a different resolution for each sample. The details of scanning X-ray microCT scanning parameters are given in Table S2. Reconstruction techniques such as beam hardening correction, defect pixel masking, and ring artifact reduction were applied using NRecon (Bruker, Belgium) to refine the raw projections. The final grayscale images were eventually thresholded using the Otsu automatic thresholding technique via CTAn (Bruker, Belgium) to produce binary images for further analysis.^25^

Different volumes of interest (VOIs) of 7–11 mm^3^ were extracted from the binary data set of each sample for computing the geometrical and physical features. The porosity of the samples was obtained using a simple voxel counting algorithm. On the other hand, the pore size distribution was analyzed by applying distance transform and local thickness algorithms within the void space. Additionally, the surface area per unit volume of the nonwoven samples was quantified by measuring the pixel area corresponding to the fibers’ outer surface using CTAn (Bruker, Belgium) and subsequently dividing it by the VOI.

### 2.3 Scanning Electron Microscopy (SEM) Analysis

The in-plane fiber orientation distribution and average fiber diameter of the nonwoven samples were determined using SEM analysis. Each nonwoven sample (∼5 × 8 mm^2^) was coated with a 10–15 nm thick gold layer using Quorum Q150R Plus sputter coater and mounted on a ThermoFisher Scientific Apreo C LoVac SEM stub with the help of an adhesive carbon tape. The samples were then examined at magnifications of 250× and 1500×, and three images per sample were taken at each magnification. The SEM images were analyzed using an open-source software, ImageJ,^26^ to measure fiber orientation and diameter.

### 2.4 Wetting Behavior

The apparent contact angle of nonwoven samples was obtained using a Dataphysics OCA 15EC goniometer. A 10 μL droplet was dispensed at a 2 μL/s dosing rate using a 25-gauge needle mounted to a Hamilton 100 μL glass syringe. The images were captured using a digital camera, and the apparent contact angles were subsequently measured from them. At least 10 measurements were recorded at various locations on the sample and averaged to determine the apparent contact angle.

### 2.5 Tensile Properties of rCFs

The tensile behavior of rCFs was obtained using an Instron 5500R universal testing machine (UTM). Individual fibers were extracted from the nonwoven samples and mounted on a C-shaped window with the help of a cyanoacrylate-based adhesive to avoid slippage at the gripping jaw. The initial gauge length was maintained to be 10 mm, and the specimen was extended at a constant rate of elongation of 0.1 mm/min.

### 2.6 Cell Culture

To study the biocompatibility of the rCF-based nonwoven materials, NIH/3T3 fibroblasts (CRL-1658, ATCC, Manassas, VA) and Calu-3 lung epithelial cells (HTB-55, ATCC) were used. NIH/3T3 cells were cultured in Dulbecco’s modified Eagle medium (DMEM, DMEM-HA, Capricorn Scientific, Germany) supplemented with 10% fetal bovine serum (FBS, FBS-11A, Capricorn Scientific) and 1% penicillin-streptomycin (P/S, P4333, Sigma-Aldrich, St. Louis, MO), while Calu-3 cells were cultured in a 1:1 mixture of DMEM/nutrient mixture F-12 (DMEM/F12, 11320033, Thermo Fisher Scientific, Waltham, MA) containing 10% FBS, 1% P/S and 1% nonessential amino acids (11140050, Thermo Fisher Scientific).

NIH/3T3 cells were passaged at approximately 60% confluence by incubation with trypsin-EDTA (T4049, Sigma-Aldrich) for 6 min until cell detachment after a prior wash step with phosphate-buffered saline (PBS, 18912014, Thermo Fisher Scientific). These cells were then transferred to a new culture flask with a splitting ratio of 1:20.

Calu-3 cells were passaged at approximately 80% confluence in two steps. After washing with PBS, cells were incubated with trypsin-EDTA for 5 min. This procedure was repeated with an incubation time of 6 min until cells detached. The suspension was then centrifuged at 400 rpm, and cells were resuspended and plated at 1:8 in a new cell culture flask.

Both cell types were cultured at 37 °C in an air-like atmosphere containing 5% CO_2_. Cell culture medium was changed every 2 days. When preparing cells for subsequent experiments, the number of cells was determined using an automatic cell counter (EVE, NanoEntek, Korea) prior to seeding.

### 2.7 Nonwoven Sample Preparation for Cell Culture

Samples A, B, and C were sterilized prior to use in contact with cells. Initially, nonwoven samples with surface areas of 0.25 and 1 cm^2^ were transferred to plastic tubes filled with 70% ethanol. For sterilization, these tubes were placed in an ultrasonic water bath (Branson 3510 Ultrasonic Cleaner, Emerson Electric, St. Louis, MO) containing water and soap at 50 °C for 15 min. Subsequently, the nonwoven samples were dried in a sterilized environment under ultraviolet (UV) light, rinsed twice with PBS, and dried again under UV light. For cell culture experiments, 1 cm^2^ nonwoven samples were used in 12-well plates (665180, Greiner Bio-One, Austria), while 0.25 cm^2^ nonwoven samples were used in 24-well plates (662160, Greiner Bio-One).

### 2.8 Fluorescent Cell Staining

For live staining of Calu-3 cells with CellTracker Green CMFDA (C7025, Thermo Fisher Scientific), cells were first rinsed with PBS after 4 days of incubation on the materials. Subsequently, nonwovens, including cells, were incubated in a solution of CellTracker Green CMFDA and DMEM/F12 with a final concentration of 10 μM for 90 min at 37 °C, followed by two washing steps with PBS and transfer to standard growth media.

The Focal Adhesion Staining Kit (FAK100, Sigma-Aldrich)—consisting of DAPI (4′,6-diamidino-2-phenylindole, 100 μL), TRITC-conjugated phalloidin (tetramethylrhodamine, 15 μg) and a vinculin monoclonal antibody (antivinculin, purified clone 7F9, 100 μL at 1 mg/mL)—was applied with the corresponding fluorescent-labeled secondary antibody (Goat anti-Mouse IgG (H+L), Alexa Fluor 488, 500 μL at 2 mg/mL, A-11001, Thermo Fisher Scientific) to visualize cell nuclei, the actin cytoskeleton and focal contacts, respectively. Prior to this, cells on the materials were fixed with a 4% formaldehyde solution (J60401-AK, Thermo Fisher Scientific) for 15 min at room temperature. Subsequently, samples were rinsed twice with a wash buffer of PBS containing 0.05% Tween 20 (P1379, Sigma-Aldrich). Thereafter, cells were exposed to a permeabilization solution consisting of 0.1% Triton X-100 (X100, Sigma-Aldrich) in PBS for a period of 5 min. The procedure continued with a 2-fold rinse with the wash buffer, followed by an incubation of cells with a blocking solution consisting of 1% bovine serum albumin (A9418, Sigma-Aldrich) in PBS for 30 min at room temperature. The blocking solution was then supplemented with antivinculin at a ratio of 1:200. Cells were incubated in this solution for 1 h at room temperature. Afterward, three washes of 5 min each were conducted with the wash buffer. The secondary antibody and the phalloidin, which had been previously resuspended in 250 μL DMSO (dimethyl sulfoxide, D12345, Thermo Fisher Scientific), were added to the materials with cells in PBS solution at a ratio of 1:1000. After incubation at room temperature for 45 min, cells were subjected to three additional 5 min washes with the aforementioned buffer. Subsequently, cells were treated with a 1:500 dilution of DAPI in PBS, incubated for 5 min, and washed three times as previously described.

### 2.9 Protein Adsorption

Samples A, B, and C were coated with rhodamine-labeled laminin (LMN01, Cytoskeleton, Denver, CO) using the following protocol. First, the laminin solution was prepared by dissolving it in the cell culture medium of the Calu-3 cells to a concentration of 4 μg/mL. The materials were then immersed in the laminin solution for 5 min at room temperature. Finally, samples were dried for 45 min at room temperature.

### 2.10 Fluorescence Imaging

Visualization of cells, cell nuclei, and corresponding proteins was performed on an Axio Observer.Z1 fluorescence microscope (Carl Zeiss Microscopy, Germany) equipped with the CSU-X1A spinning disk unit (Yokogawa Electric, Japan). An EC Plan-Neofluar 5×/0.16 Ph1 M27 objective (4203319911000, Carl Zeiss Microscopy) was used at 5× magnification and an EC Plan-Neofluar 10×/0.3 Ph1 M27 objective (420341991100, Carl Zeiss Microscopy) at 10× magnification. For 25× magnification, an LCI Plan-Neofluar 25×/0.8 Imm Corr Ph2 M27 immersion objective (4208519972000, Carl Zeiss Microscopy) with glycerine was used. Solid state lasers with wavelengths of 405, 488, and 561 nm were required for the excitation of the different fluorescent dyes. Here, rhodamine labeled laminin was examined using the 561 nm solid state laser and the 10× objective, while CellTracker Green was excited with the 488 nm solid state laser and imaged with the 5× objective, respectively. Excitation of DAPI, TRITC-phalloidin, and the secondary antibody was performed with the 405, 488, and 561 nm solid state lasers, respectively, and imaging was carried out using the 25× magnification objective. All images were taken with an exposure time of 200 ms. An Orca Flash 4.0 camera (Hamatsu Photonics, Japan) was used to digitize the images, which were processed with the Zen 2.6 blue edition (Carl Zeiss Microscopy) afterward. In this experimental setup, 12-well plates with 1 cm^2^ nonwoven samples were used. During imaging, cell culture atmosphere and temperature were maintained.

### 2.11 Cell Viability and Proliferation Assay

To investigate viability and proliferation of cells in contact with the nonwoven samples A, B and C, luminescence measurements were performed on a Microplate Reader Synergy H1 (Agilent Technologies, Santa Clara, CA) using the CytoTox-Glo Cytotoxicity Assay (G9292, Promega, Madison, WI). All three previously mentioned nonwovens, with a size of 0.25 cm^2^, were used. For the assay, Calu-3 and NIH/3T3 cells were seeded on nonwovens at varying densities and for distinct periods in 24-well plates. For the Calu-3 cells, the number of seeded cells was 82,000 per well, while for NIH/3T3 cells, it was 14,000, respectively. For both cell types, the number of seeded cells was halved for the eight-day period culture experiment in order to avoid cell overcrowding.

In order to ascertain the viability of the cells and to calculate their proliferation rate in a control group without contact with the nonwoven materials, both cell lines were seeded into cell culture wells only filled with cell media. Cell behavior in the respective wells was examined immediately after seeding, after 4 days, and after 8 days, respectively. Cell numbers were determined using different procedures: (1) The nonwoven sample was transferred to a new well with a fresh medium shortly before the cell number measurement to ensure that only cells on or within the nonwoven were counted. (2) Finally, a group with medium initially in contact with nonwovens for 24 h was measured. In this case, the different nonwovens were soaked in the cell culture medium for 24 h. Subsequently, the nonwoven samples were removed, and both cell lines were cultured in the medium previously exposed to the nonwovens for 4 days to assess potential toxicity from the materials or any released ions.

All groups with an eight-day growth period underwent a medium change after 4 days of incubation. For the four-day growth period, there was no medium change between days. The wells contained 500 μL of the respective cell culture medium.

In the measurement protocol for cell proliferation, the well plates were initially placed in the plate reader, and the luminescence signal of the individual wells was measured as a background signal. The luminescence was determined at a readout height of 1 mm and an integration time of 1 s. Subsequently, 350 μL of CytoTox-Glo (AAF-Glo Substrate, G607A, Promega) was added to the wells, and the well plate was returned to the plate reader, where it was shaken for 2 min at an angular velocity of 807 cpm and then incubated for 15 min at room temperature. The luminescence was measured to obtain the signal of the apoptotic cells. In the third step, 350 μL of lysis reagent, consisting of a ratio of 5 mL assay buffer (G146A, Promega) and 33 μL digitonin (G944A, Promega), was added to kill the remaining living cells. The well plate was shaken again for 2 min and incubated for 15 min, after which the luminescence was measured to obtain a signal for all cells. All data were acquired using the BioTek Gen5 software (Agilent Technologies).

Viability and proliferation percentages were calculated by comparing the luminescence signal measured at different assay stages and between different experimental groups, with the background signal being subtracted in this process.

### 2.12 Statistics

To evaluate cell attachment and protein adsorption on the nonwovens, fluorescence imaging was conducted on three sample regions for each material, allowing visualization of stained cells and laminin adsorption. For cell viability and proliferation data, 20 measurements were conducted, with six wells per group serving as data points. One data point describes the average of 21 individual luminescence measurements over the entire well, which resulted in a total of 240 wells being analyzed (see Table S3).

Data analysis and statistical analysis, as well as the creation of plots, were carried out using Python (see Supporting Information). The statistical tests utilized to compare multiple groups were either ANOVA with Tukey’s posthoc test, Welch’s ANOVA with the Games-Howell posthoc test, or the Kruskal–Wallis H-test with the pairwise Mann–Whitney U-test including Bonferroni adjustments as a posthoc test, depending on whether the respective conditions were met. Two groups were analyzed using either an independent Student’s *t*-test with or without Welch’s correction or a Mann–Whitney U-test, depending on the fulfillment of the requisite conditions. Prior to analysis, the assumptions of normality and homogeneity or heterogeneity of variances were confirmed or rejected using the Shapiro-Wilk test and Bartlett’s test, respectively, with a significance level of 0.05. Further, all figures underwent revision using Adobe Illustrator and Adobe Photoshop (see Supporting Information). The notable significant differences in the results are designated as follows: *p* < 0.05 (*), *p* < 0.01 (**), and *p* < 0.001 (***). The precise p-values can be found in the Supporting Information (see Tables S4 and S5).

## 3 Results

### 3.1 Morphological Analysis

**Figure 2:**
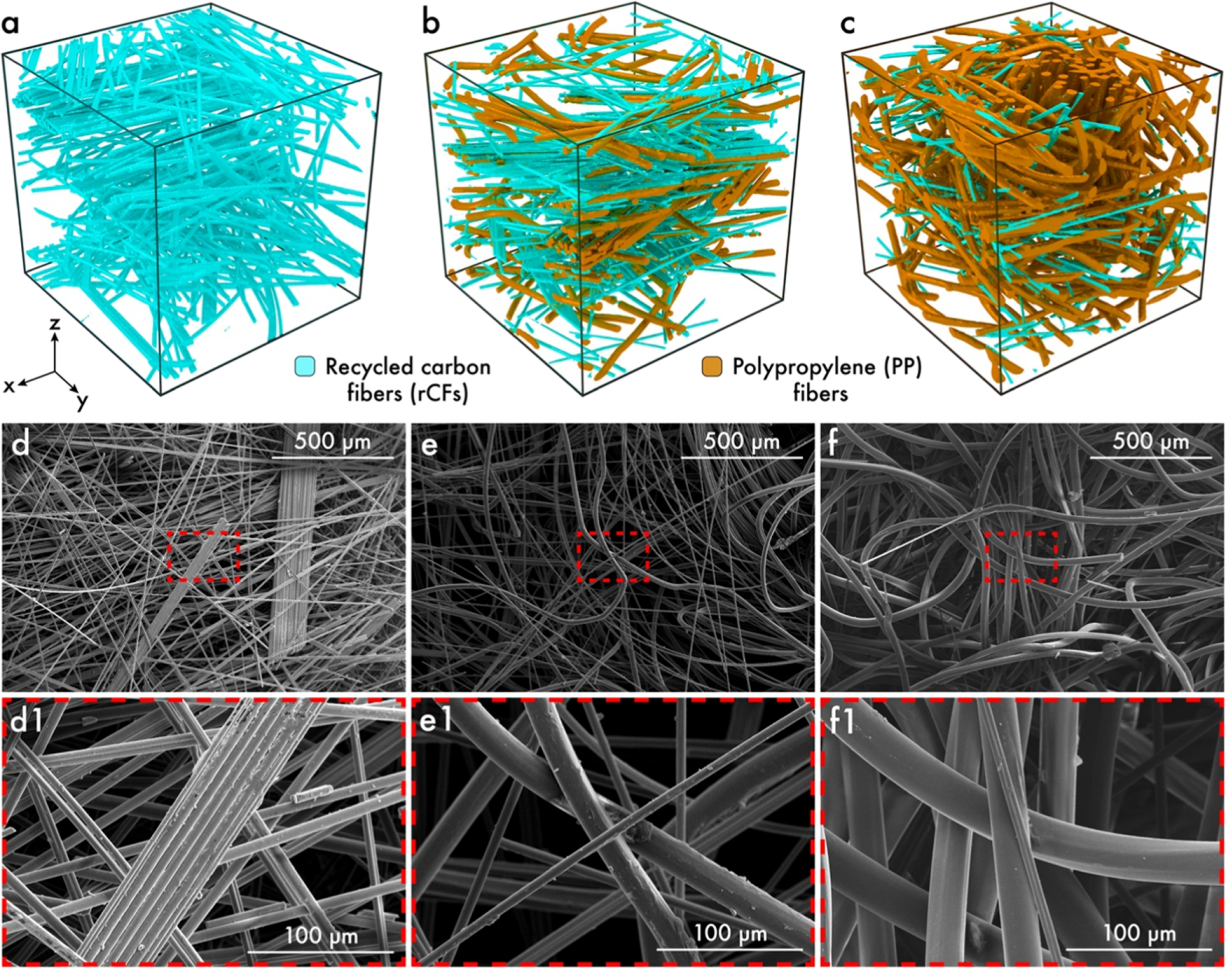
3D rendered images of samples (a) A; (b) B; and (c) C obtained via X-ray microCT data. SEM images of the sample (d), (d1) A; (e), (e1) B; and (f), (f1) C. Here, the SEM images depicted in panels (d), (e), and (f) were acquired at a magnification of 250×, whereas the images in panels (d1), (e1), and (f1) were captured at 1500× magnification.

X-ray microCT has been leveraged to reveal the spatial distribution of rCFs and PP fibers in samples A, B, and C, as depicted in Figures 2a–c and S1. As aforementioned, sample A is composed exclusively of rCFs, while samples B and C comprise a blend of rCFs and PP fibers. Generally, the aggregates or bundling of rCFs have been observed through X-ray microCT analysis, as also exemplified by the SEM images of sample A (see Figure 2d,d1). Such bundles predominantly occur due to unopened carbon fiber rovings during the processing of the fibrous web via carding.^27,28^ Nevertheless, sample B, which comprises more than 50% rCFs, exhibits a nonuniform distribution of rCFs and PP fibers, as shown in Figure 2b,e. In contrast, sample C, with lower rCFs content, shows a relatively uniform distribution of rCFs in the nonwoven, as shown in Figure 2c,f.

Nonwoven materials with a high surface area-to-volume ratio can emulate the biological environment, thereby replicating the function of native tissues.^29^ In this work, a high surface area-to-volume ratio of the nonwoven material, ranging between 140 and 200 cm^2^/cm^3^, has been computed, aligning well with values reported in the literature (see Table S1).^30^ Given the smaller fiber diameter, the rCFs have a significantly larger specific surface area (0.317 m^2^/g) compared to PP fibers (0.146–0.219 m^2^/g), which can facilitate better cell attachment. Notably, the maximum pore size of the prepared nonwoven samples remains within a range of 229–372 μm (see Table S1 and Figure S2). Such a variation in pore size arises from distinct differences in porosity and diameters of rCFs and PP fibers, as shown in Table S1.

**Figure 3:**
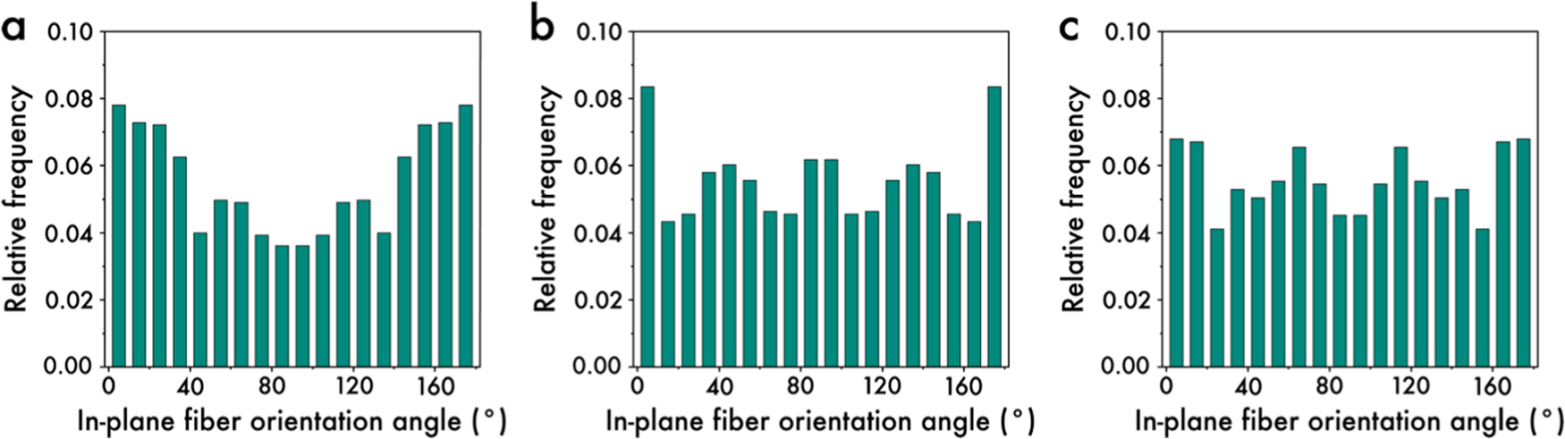
In-plane fiber orientation distribution of samples (a) A, (b) B, and (c) C obtained from SEM images. Here, 0° is the cross-machine direction, and 90° is the machine direction.

In this work, the fibers in the needlepunched nonwovens are anticipated to be preferentially aligned in the cross-machine direction, i.e., normal to the production direction. Counterintuitively, samples B and C exhibit a quasi-random structure, while sample A demonstrates a preferential alignment of constituent fibers in the cross-machine direction (see Figure 3a–c). The barbed needle, designed for finer rCFs, may not adequately accommodate the PP fibers, leading to some being released during transportation. Consequently, fibers initially oriented in the cross-machine direction may realign in the machine direction during their release.^31^ Nevertheless, the orientation of fibers has been quantified via metrics derived from the orientation averaging, i.e., *f*_p_ (=2⟨cos^2^ φ⟩ – 1) and *g*_p_ (=(8⟨cos^4^ φ⟩ – 3)/5) where ⟨cos^*q*^ φ⟩ = ∫_0_^π^ cos^*q*^ φΩ(φ)dφ (*q* = 2,4), and  is the in-plane fiber orientation distribution function, where *N*(φ) represents the number of fibers that fall within a bin of width, Δφ.^32^ Both *f*_p_ and *g*_p_ are normalized to zero and unity corresponding to a random planar distribution and perfect alignment in the uniaxial direction, respectively.

Intermediate values of *f*_p_ and *g*_p_ represent various partial states of fiber orientation. Higher powers of ⟨cos^2^ φ⟩ eliminate the possibility of various types of in-plane fiber orientation distributions in nonwoven materials.^32^Table S1 shows the values of *f*_p_ and *g*_p_ indicating that sample A has the preferential alignment of fibers in the cross-machine direction followed by samples B and C indicating their “quasi-isotropic” nature. Further, the fiber orientation characteristics have been ascertained by the comparable magnitudes of *f*_p_ and *g*_p_, as shown in Table S1.

### 3.2 Wetting Behavior

**Figure 4:**
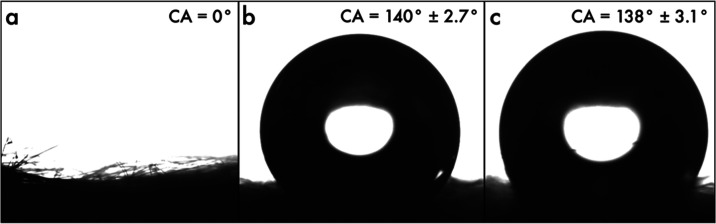
Images obtained for determination of static apparent contact angle of samples (a) A, (b) B, and (c) C. The average static apparent contact angle was calculated from 5 observations.

Designing biomaterials focusing on wettability could better investigate biological liquid environments and regulate associated cellular behaviors, thereby advancing the development of biomaterials and biomedicine.^33^ Hydrophilic ECM proteins, such as laminin, show a strong affinity for hydrophilic implant materials, which may be due to their elevated surface energy.^34^ In this study, laminin was dissolved in the hydrophilic cell culture medium, DMEM, further augmenting its affinity for wettable surfaces. The wettability of the nonwoven samples has been assessed by determining the apparent contact angles with water, as depicted in Figure 4.

Sample A, exhibiting a contact angle of 0°, herein is classified as superhydrophilic due to the rapid spreading of water on the nonwoven surface of rCFs (see Figure 4a and Movie S1). Carbon fibers are typically treated with sizing materials to enhance processability, which remains even after recycling and modify the inherent wettability of carbon fibers from hydrophobic to hydrophilic.^35^ On the other hand, the addition of moderately hydrophobic PP fibers in samples B and C tends to enhance the apparent contact angles, as depicted in Figure 4b,c, and Movie S1. Notably, the wettability of a surface is governed by its roughness and the inherent nature (surface energy) of the solid.^36^ Roughness in nonwoven materials arises from protuberances created by overlapping fibers in defined regions.^37^ In sample A, the hydrophilicity of rCFs has been amplified by the roughness of the nonwoven material, while this roughness augments the hydrophobicity in both samples B and C. Thus, the nonwoven roughness amplifies the hydrophilicity of rCFs in sample A and enhances the hydrophobicity in both samples B and C. As aforementioned, sample B comprises the bundling of carbon fibers within the nonwoven structure, resulting in a nonuniform distribution of rCFs and PP fibers. This uneven arrangement likely increases the exposure of hydrophobic PP fibers to water, reducing the hydrophilic contribution of the rCFs. According to the Cassie–Baxter model,^38^ this uneven distribution can trap air at the interface, further amplifying the hydrophobicity of the surface despite the presence of hydrophilic carbon fiber. In other words, the bundling of carbon fibers in sample B reduces their effective solid–liquid contact area (*f*_1_) in Cassie–Baxter’s model,^38^ expressed as cos θ_CB_^*^ = *f*_1_ cos θ_*Y*_ – *f*_2_, where θ_*Y*_ is the equilibrium contact angle determined on the smooth surface in accordance with Young’s equation, θ_CB_^*^ is the apparent contact angle of a composite interface, and *f*_1_, *f*_2_ are the area fractions of the solid/liquid and liquid/air, respectively. This reduction in *f*_1_ leads to hydrophobic behavior comparable to that of sample C.

### 3.3 Protein Adsorption

**Figure 5:**
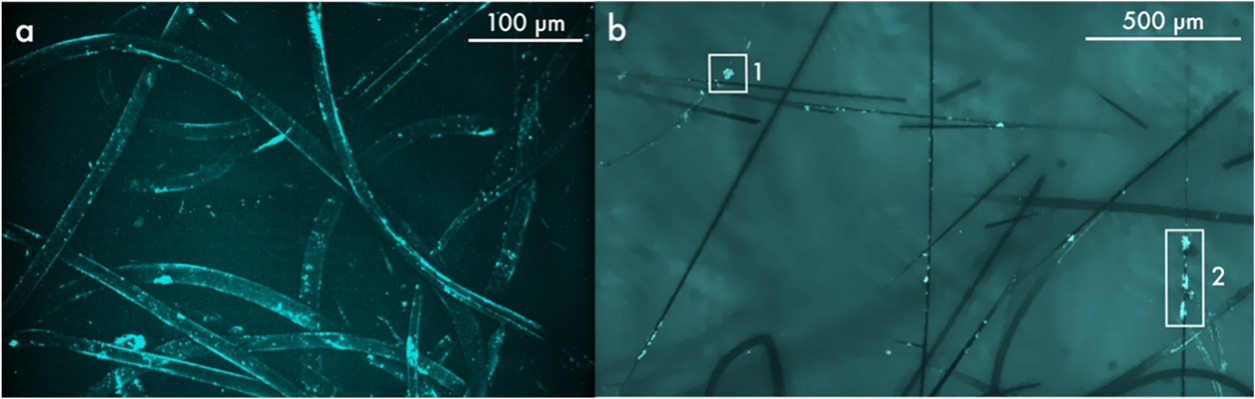
Fluorescence microscopy images of a (a) *z*-projection of a 631.5 μm thick *z*-stack of nonwoven sample C without fluorescent labeling at a magnification of 10× and an excitation wavelength of 561 nm. As a consequence of the *z*-projection, only PP fibers are discernible. They display a luminescence resulting from total internal reflection and scattering of the laser light on the translucent PP fibers. (b) A single *z*-plane of a 122.1 μm thick *z*-stack of nonwoven sample B treated with 4 μg/mL rhodamine-labeled laminin at a magnification of 10× and an excitation wavelength of 561 nm is shown. The thinner black fibers can be identified as rCFs, whereas the thicker fibers correspond to PP fibers. The PP fibers only exhibit their characteristic luminescence within the focal plane (see vicinity of region 2). In region 1, a laminin cluster is displayed on what is likely a PP fiber, while region 2 shows multiple laminin clusters attached to a rCF. Additionally, beyond these marked regions, there are numerous smaller laminin clusters in turquoise, primarily on rCFs.

Biocompatibility of a material often correlates with the potential for proteins present in the ECM to adsorb onto the surface of the respective biomaterial. These proteins, secreted by the cells, enhance their ability to adhere to surfaces, spread, and proliferate. Thus, in the first step, we soaked the nonwoven materials in the respective cell culture medium containing rhodamine-labeled laminin, a common protein from the ECM important for the formation of focal adhesion sites. Here, Figure 5a presents an image of nonwoven sample C, which did not undergo coating with rhodamine-labeled laminin. In Figure 5b, however, nonwoven sample B was imaged subsequent to the application of rhodamine-labeled laminin. Upon examination under a fluorescence microscope, the rCFs were characterized by thin, straight, black fibers, whereas the PP fibers were more curved and thicker. As a result of utilizing a projection of a 631.5 μm thick *z*-stack in Figure 5a, the visualization of any rCFs, despite their 10% contribution, is not discernible. Moreover, PP fibers exhibited characteristic luminescence due to scattering and total internal reflection in the focal plane, as evidenced in Figure 5a and in the vicinity of region 1 in Figure 5b. As illustrated in Figure 5b, not all PP fibers show this luminescence since the signal becomes only present in the focal plane, as shown in Figure 5a. Consequently, the nonluminous regions of the fibers were not situated within the area of focus. Following the treatment of the nonwovens with rhodamine-labeled laminin, the adhesion of the laminin to rCFs was observed in the form of individual clusters, as can be seen in region 2 of Figure 5b. In the laminin-treated sample B depicted in Figure 5b, laminin clusters may adsorb on the PP fibers; however, the adsorption of laminin on PP fibers was difficult to image because of the scattering and total internal reflection concealing possible signals from the laminin (region 1). Nevertheless, based on visual inspections, we can conclude that laminin adsorption on PP fibers was sporadic, whereas laminin adsorption on rCFs was more prevalent (see Figure S3).

### 3.4 Cell Organization

**Figure 6:**
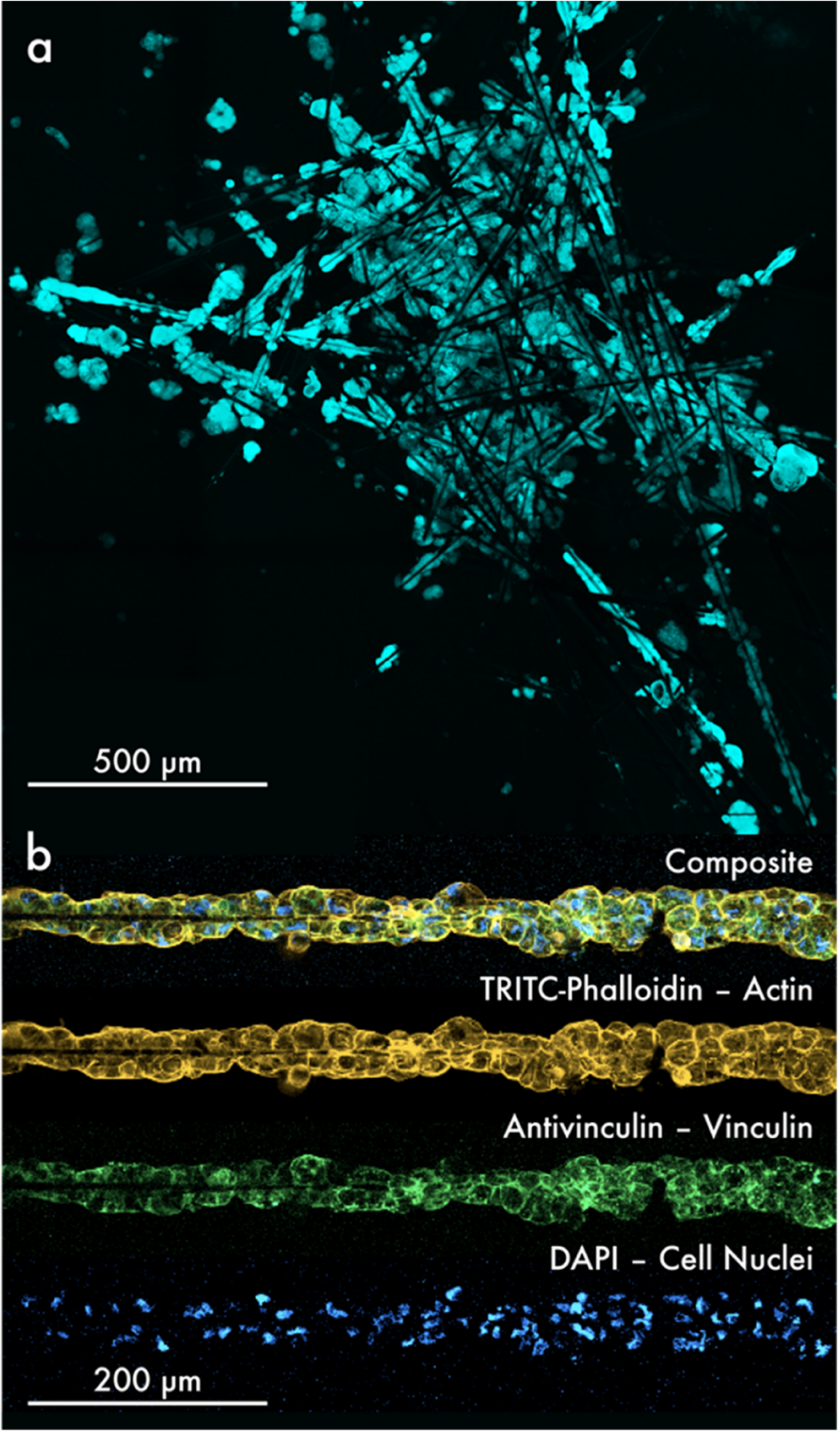
(a) Cell organization on nonwovens. Projection of a z-stack with a total thickness of 63.15 μm, imaged with a fluorescence microscope. The cell cluster, consisting of Calu-3 cells attached around rCFs in a nonwoven sample A, was stained with CellTracker Green CMFDA (10 μM) and imaged at a magnification of 10×, at an excitation wavelength of 488 nm. (b) Magnification of cells adhering to a rCF of a nonwoven composed of 100% rCFs. The fluorescence image depicts the individual channels of the stained structures, namely actin, vinculin, and the cell nuclei, which were stained with TRITC-phalloidin, antivinculin, and DAPI, respectively, in Calu-3 cells surrounding a single rCF. The top image of Figure 6b illustrates the union of all channels and was captured with a magnification of 25× and lasers at 405, 488, and 561 nm, respectively.

Cell organization was investigated by utilizing Calu-3 cells on the aforementioned materials. Here, we employed lung epithelial cells to study the applicability of carbon-based nonwoven materials as artificial lung scaffolds. As illustrated in Figure 6a, cells were investigated in contact with the nonwoven sample A. In comparison, Figure 6b demonstrates the attachment of cells to a singular rCF. Following an incubation period of 4 days, we observed that Calu-3 cells had uniformly dispersed across the entire nonwoven structure, demonstrating adherence to the rCFs. Figure 6a,b illustrate that, in the case of a single rCF, cells formed clusters along the fiber. However, the overlapping of multiple rCFs (see Figure 6a) resulted in the merging of the Calu-3 cell clusters along the fibers, forming a large conglomerate on and between the rCFs. Notably, no cell adhesion to PP fibers could be observed after 4 days of incubation. Nevertheless, the use of the two other nonwovens, samples B and C, also resulted in the adhesion of numerous Calu-3 cells to the rCFs (see Figure S4).

By examining the Calu-3 cells along a single rCF as shown in Figure 6b, cell organization and adhesion could be investigated. The Calu-3 cells were observed to adhere closely together along a 600 μm (approximately) long section of an rCF in a nonwoven sample A, forming a dense and compact cluster with no free spaces visible between the cells. The Calu-3 cells are wrapped around the rCF, exhibiting normal morphology and positioning of their nuclei and actin cytoskeleton. Additionally, the protein vinculin, important for the formation of cellular focal contacts,^39^ is observed to be particularly prominent at the edge of the cells. Furthermore, the examination of individual fibers revealed no cell adhesion to PP fibers, consistent with previous observations that protein adsorption—a crucial step for cell adhesion—was predominantly observed on rCFs. Adhesion of cells to rCFs of the other nonwovens—samples B and C—also exhibited a comparable behavior, as illustrated in Figure S5.

### 3.5 Cell Viability

The biocompatibility of the nonwovens with different cell lines was assessed by quantifying the percentage of living cells and measuring cell viability at four- and eight-day intervals.

**Figure 7:**
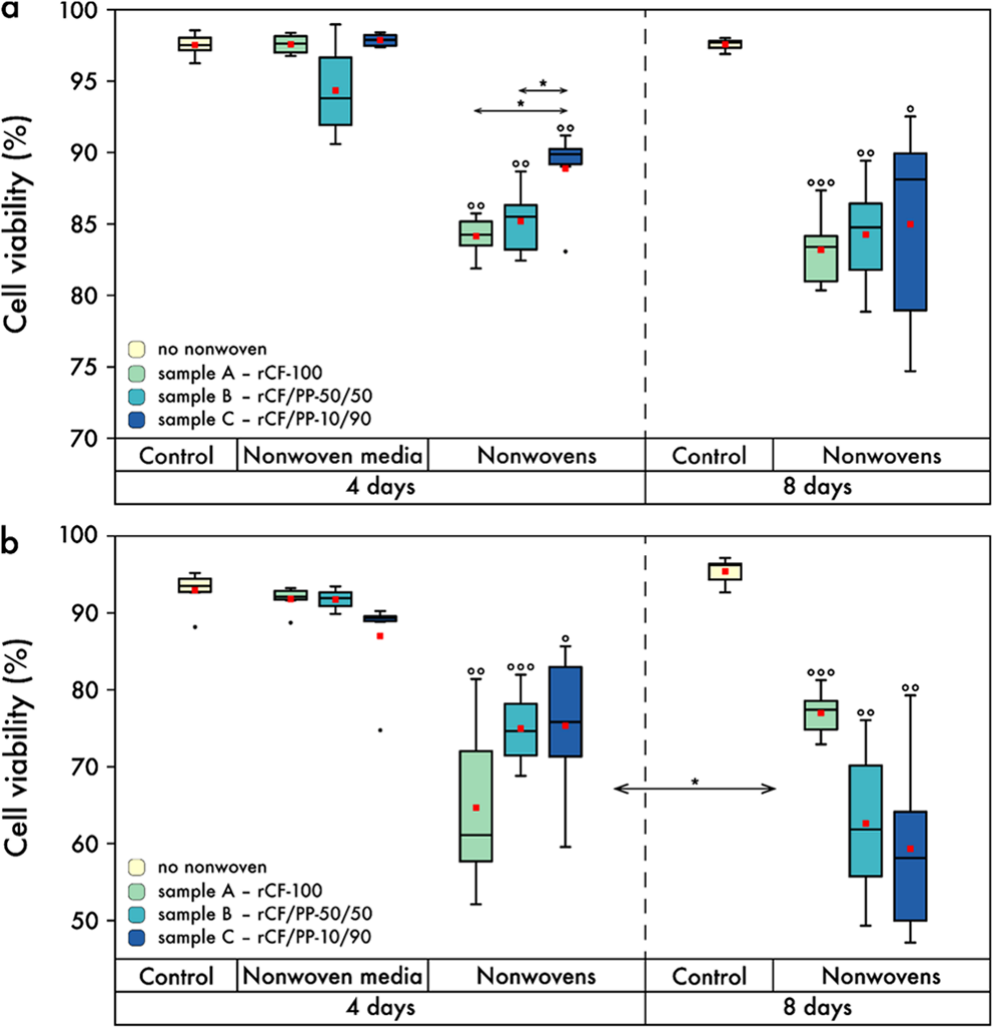
Boxplot of cell viability, viz. living cells, of (a) NIH/3T3 fibroblasts and (b) Calu-3 lung epithelial cells. The data represents the results of experiments conducted under varying growth conditions. Cells were cultured on nonwovens materials (labeled as ’Nonwovens’), or alternatively in nonwoven-exposed medium (labeled as ’Nonwoven media’), for a period of 4 or 8 days, respectively. Additionally, cells were cultivated under ideal cell culture conditions (labeled as ’Control’) for both periods. Red squares indicate mean values, while central lines represent median (50th percentile). The bottom and top of the box (hinges) represent the 1st and 3rd quartiles, respectively. The end of the whiskers represents the minimum and maximum of the data (hinges ± 1.5 interquartile range). Black dots serve to indicate outliers. Colors represent the specific nonwovens utilized: pale yellow (cream)—no textile; pale green (celadon)—sample A; pale cyan (moonstone)—sample B; blue (YInMn blue)—sample C. Significances in comparison to the control group are indicated with circles, while significances within the groups are marked with asterisks. The following significance levels are used: °/*: *p* < 0.05, °°/**: *p* < 0.01, °°°/***: *p* < 0.001.

Figure 7a illustrates the outcomes observed with NIH/3T3 cells. To provide a point of reference, a control experiment was conducted without nonwovens under idealized cell culture conditions, wherein cell viability after 4 and 8 days was found to be (97.5 ± 0.3)% and (97.6 ± 0.2)%, respectively, for the NIH/3T3 cells. This indicates that the vast majority of cells survived.

To investigate possible cytotoxic effects of the nonwovens on the cell culture medium, NIH/3T3 cells were cultivated for 4 days in nonwoven-soaked medium (see Methods, Cell Viability and Proliferation Assay). The viability of NIH/3T3 cells (see Figure 7a) on samples A, B, and C was (97.6 ± 0.3)%, (94.4 ± 1.4)%, and (97.9 ± 0.2)%, respectively. These values were found to be comparable to those of the control group and not significantly different. The exact p-values can be found in Table S4.

Finally, cell viability was assessed after a four-day period by culturing NIH/3T3 cells directly on the nonwovens. The measured viabilities of (84.1 ± 0.6)%, (85.2 ± 1.0)%, and (88.9 ± 1.2)% for nonwoven samples A, B, and C, respectively, demonstrated statistically significant differences when compared to the control group (marked by the circles), as well as to each other (marked by asterisks). In comparison with the control group, results demonstrated statistical significance at a significance level of *p* < 0.01. For samples A and B, as well as samples A and C, statistically significant differences were observed with *p* < 0.05. A comparison of NIH/3T3 cell viability on nonwoven samples over the course of 4 days and 8 days revealed no statistically significant differences. The viabilities on samples A, B, and C after 8 days were (83.2 ± 1.1)%, (84.3 ± 1.6)%, and (85.0 ± 3.1)%, respectively. These values exhibited notable discrepancies from the eight-day control measurement, as illustrated in Figure 7a. The respective statistical significance levels associated with these observations are delineated in the caption.

A comparable picture emerged for the Calu-3 cells in Figure 7b, with cell viabilities approaching 100% for the control measurements at 4 days [(92.9 ± 1.0)%] and 8 days [(95.4 ± 0.7)%]. As previously observed, the group cultured in the nonwoven-exposed medium for 4 days demonstrated high cell viability and no notable disparities from the control group, with viabilities of (91.8 ± 0.7)%, (91.7 ± 1.0)%, and (87.0 ± 2.5)% for samples A, B, and C, respectively.

The trend observed in the Calu-3 cells in Figure 7b was essentially analogous to that observed in the NIH/3T3 cells in Figure 7a. Similarly, the cell viabilities on the nonwoven samples after 4 days were diminished in comparison to the control, yet remained relatively high [A: (64.6 ± 4.6)%, B: (75.0 ± 2.0)%, C: (75.3 ± 4.0)%]. Therefore, notable discrepancies were evident between each material and the control, as indicated by the corresponding symbols in Figure 7b. However, a significant difference (*p* < 0.05) was observed between the cell viabilities of each material after 4 days and its respective counterpart after 8 days using the Calu-3 cells [A: (77.0 ± 1.3)%, B: (62.6 ± 4.2)%, C: (59.4 ± 4.9)%]. The cell viability of the nonwoven-based textiles group after 8 days was partly reduced compared to the eight-day control group, thereby demonstrating a statistically significant difference. The observed increase in viability from 4 to 8 days for sample A can be attributed to the presence of sufficient surface area and available adhesion sites, which facilitate the proliferation and migration of the Calu-3 cells. Here, the bundling of rCFs reveals close proximity between them, often resulting in overlapping configurations (see Figure 2d,d1). Similarly, samples B and C demonstrate an adequate surface area and a sufficient number of vacant adhesion sites. However, these uncolonized rCFs are too distant and separated from the already colonized rCFs due to the high PP fiber content, which impedes the cells’ ability to access these uncolonized areas. Consequently, during the growth period from 4 to 8 days, the cell density of the overgrown rCF areas is so high that, due to a lack of space and cell crowding, the total number of living cells no longer increases. This results in a decrease in viability for the 50/50 and 10/90 rCF-to-PP fiber blends in comparison to 4 days.

### 3.6 Cell Proliferation

**Figure 8:**
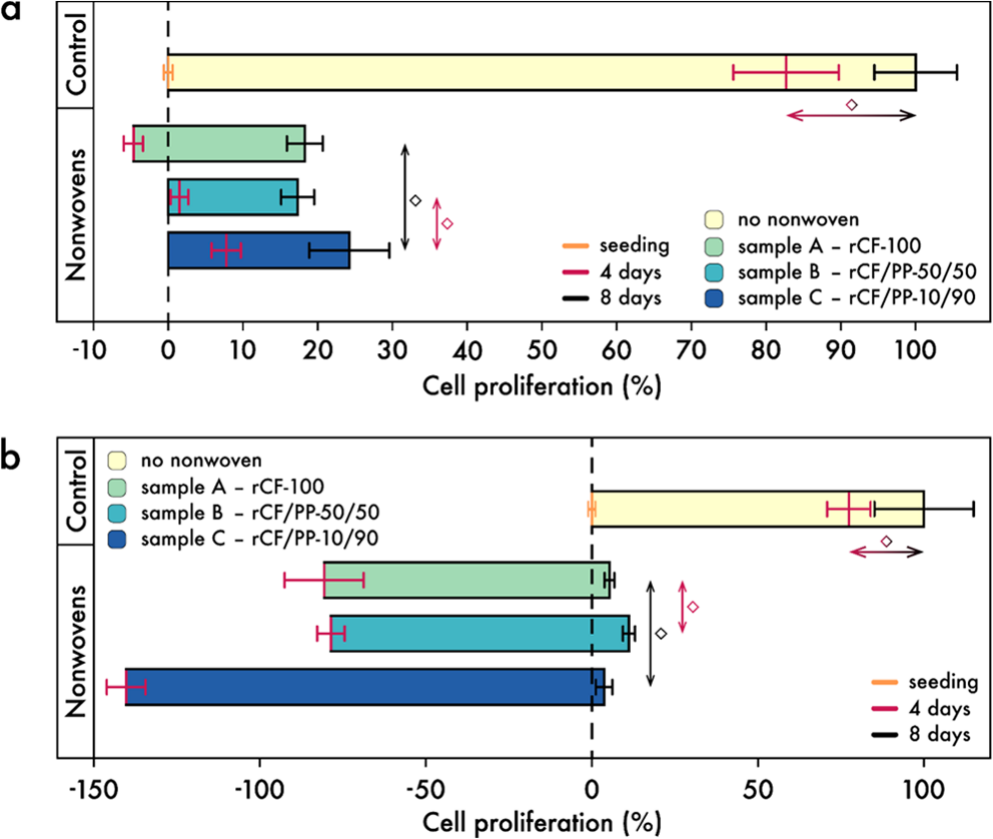
Cell proliferation of (a) NIH/3T3 fibroblasts and (b) Calu-3 lung epithelial cells. The data were normalized so that 0% proliferation equaled the control outcome at zero days after seeding, and 100% proliferation scaled to the control outcome at 8 days after seeding. Cells were cultured on nonwoven materials (labeled as ’Nonwovens’) or under idealized cell culture conditions (labeled as ’Control’) for 4 or 8 days, respectively. Colors of the lines and their associated error bars indicate the days of growth: orange—zero days; red—4 days; black—8 days. Colors of the bars display the specific nonwovens utilized: pale yellow (cream)—no nonwoven; pale green (celadon)—sample A; pale cyan (moonstone)—sample B; blue (YInMn blue)—sample C. The diamond symbol indicates the absence of significance. Horizontal double arrows facilitated comparison between different growth periods, while vertical double arrows compared different materials according to the respective growth periods (see color coding).

In addition to examining survival rates of cells present on or within the nonwovens, we investigated cell division and proliferation rates and compared these between the control and experimental groups at the four- and eight-day time points. To achieve this, luminescence signals from cells with and without nonwovens, cultured for different periods, were analyzed to determine their growth rate (see Figure 8).

It is essential to highlight that the cell count immediately after seeding (illustrated by the orange lines with associated error bars) was normalized with a growth of 0%, and the growth of the cells after 8 days (depicted by the black lines with associated error bars) in the control group was scaled to 100%. All other values pertaining to the nonwoven groups and the four-day measurements (shown by the red lines with associated error bars) of the control group were normalized in accordance with the aforementioned procedure. To ensure comparability between the four- and eight-day measurements, given that different cell counts were utilized in the experimental procedure, four-day measurements of the control group with varying cell counts were employed as a reference to link these two groups.

Figure 8a illustrates the outcomes for the NIH/3T3 fibroblasts. In the control group, partially normalized growth rates were obtained for 0, 4, and 8 days, with values of (0.0 ± 0.7)%, (82.7 ± 7.2)%, and (100.0 ± 5.7)%, respectively. A closer examination of the proliferation results over 4 days reveals that the growth rates on nonwoven samples A, B, and C are notably low or even negative, viz. cell counts lower than the originally seeded cell number on the nonwovens, in comparison to the control, with values of (−4.7 ± 1.5)%, (1.5 ± 1.4)%, and (7.7 ± 2.1)%, respectively. However, a comparison of the growth rates on the nonwoven materials after 4 days with those after 8 days [A: (18.3 ± 2.6)%, B: (17.3 ± 2.4)%, C: (24.2 ± 5.5)%] reveals a notable increase for all materials, although proliferation remains considerably lower compared with the eight-day control.

In view of the diversity of significant relations between the groups, only those relations are generally shown in Figure 8, where no significant difference was observed (diamonds). For a comprehensive overview of the *p*-values, please refer to the Supporting Information, as illustrated in Table S5. In Figure 8, the horizontal double arrows indicate a comparison between the four- and eight-day periods. Significant differences were observed between the two periods for all nonwoven samples, with the exception of the control group. In the vertical direction, the nonwovens are compared with each other after 4 days (red double arrows) or after 8 days (black double arrows). It is evident that after 4 days, the proliferation values of all nonwovens exhibited a notable divergence from the control group, and, in certain instances, from one another. Nevertheless, there was no discernible difference in proliferation between samples B and C at the four-day mark. At the eight-day interval, a comparable trend emerged, with a statistically significant divergence from the control group and a nonsignificant divergence between samples A and C.

As illustrated in Figure 8b, the proliferation rates of the Calu-3 cells were observed to be (0.0 ± 1.0)%, (77.4 ± 7.0)%, and (100.0 ± 15.4)% for the control measurements at 0, 4, and 8 days, respectively. Moreover, the growth of cells on the nonwovens after a four-day period was nonexistent, given that all materials exhibited a reduction in cell count compared to the initial seeding. This is reflected in the negative growth rates of (−80.6 ± 12.3)%, (−78.6 ± 4.6)%, and (−140.3 ± 6.3)% for nonwoven samples A, B, and C, respectively. However, there was a notable recovery in comparison to the eight-day period [A: (5.3 ± 1.9)%, B: (11.2 ± 2.2)%, C: (3.7 ± 2.9)]. While the recovery is also evident in the NIH/3T3 cells in Figure 8a, it is more pronounced in Figure 8b. As a consequence, there is a notable discrepancy in cellular proliferation of Calu-3 cells across all samples after 4 and 8 days. Nevertheless, cell proliferation on the nonwovens after 8 days remains considerably lower than that observed in the control group. Furthermore, the cell proliferation of the control does not exhibit notable differences between 4 and 8 days. All nonwovens demonstrate substantial discrepancies after 4 days in comparison to the control and to one another, except for samples B and C. For 8 days, this phenomenon persists, with the exception of samples A and C.

The initially very low proliferation values can be attributed to the large pores of the nonwovens with diameters exceeding the size of the cells. To this end, during cell seeding, several cells dropped through the pores and were sinking to the bottom of the well resulting in a reduced cell number of adherent cells compared to the control group of cells seeded into well plates. These differences in cell numbers are reflected by the partly negative values observed after 4 days. Thus, it is more meaningful to examine the increase in proliferation from 4 to 8 days in conjunction with the viability values of the two cell lines on the nonwovens. Here, a notable increase of the proliferation from 4 to 8 days is observed, comparable to the control, with consistently high viabilities, which indicates a very good proliferation behavior of both cell lines on the nonwovens.

## 4 Discussion

The utilization of biomaterials in the field of tissue engineering represents a promising interdisciplinary approach to the reconstruction of tissue structures and the construction of artificial organs.^1,2^ Lung tissue represents a key area of application, as lung diseases—including lung cancer, COPD, pneumoconiosis, and pulmonary fibrosis^10,11^—are major contributors to global morbidity and mortality.^40^ To address lung damage, 3D scaffolds made from biomaterials are implanted into the lung,^41^ which are designed to stimulate cellular growth, provide a high specific surface area, and mimic the structure and morphology of lung tissue.^18^ Consequently, nonwoven materials selected for this investigation—comprising rCFs and PP fibers—demonstrated favorable characteristics for lung implants since these materials can be constructed with tunable pore sizes, a considerable surface-to-volume ratio, versatile surface functionalities, and excellent mechanical properties.^42^ Their use offers a promising alternative to conventional lung transplantation, which is limited by a shortage of available donor organs, risks of clotting complications, infections, and rejection of the transplanted lung.^43^

Accordingly, in our study, the nonwovens’ biocompatibility was evaluated with the aim of expanding upon their aforementioned promising material properties for use in tissue engineering. Ostensibly, hydrophilic laminin was effectively adsorbed onto rCFs, whereas adsorption onto hydrophobic PP fibers was either absent or negligible. Typically, carbon fibers exhibit hydrophobic characteristics,^44^ but to enhance processability, they are treated with sizing materials that remain stable even after recycling, transforming them into hydrophilic materials.^35^ Laminin, a protein constituent of the ECM,^34^ also exhibits hydrophilic properties and, like the majority of ECM proteins, displays a preference for hydrophilic surfaces.^34^ The observed phenomenon of adsorption on rCFs, with minimal or no evidence of such on PP fibers, aligns with the anticipated outcomes.^45^ Nevertheless, the potential influence of additional factors, such as the roughness and unevenness of rCFs, cannot be ruled out, as these properties directly impact the adsorption of ECM proteins.^46,47^ However, these observations of laminin adsorption are consistent with findings related to Calu-3 cell attachment to the rCFs in the scaffolds, suggesting that the nonwovens exhibit favorable bioactivity.

As previously noted, Calu-3 cell adhesion was observed exclusively on rCFs and not on PP fibers. This finding was consistent across all nonwoven blends, despite samples B and C demonstrating hydrophobic behavior. However, cell attachment to these materials does occur, as the hydrophilic rCFs serve as adhesion sites for the Calu-3 cells at the microscopic level. Further, the adsorption of laminin indicates that the adhesion of other ECM proteins enabled Calu-3 cells to adhere to the hydrophilic rCFs. Conversely, cell adhesion was prevented by the hydrophobicity of the PP fibers.^48,49^ It is noteworthy that in other studies, the adhesion of cells to hydrophobic surfaces was observed, albeit in lower cell numbers and with less efficacy than on hydrophilic surfaces.^48,50^ Given that the observations of cell attachment were only conducted after 4 days, it is plausible that Calu-3 cells may have adhered to the PP fibers within the initial few hours. Due to the limited adhesion to hydrophobic surfaces and the relatively large pores of the nonwovens, only a few isolated cells likely adhered to the PP fibers. This could have resulted in a deficit of cell–cell contacts and a deficiency of growth factors, which ultimately precipitated apoptosis or anoikis over the course of 4 days.^51,52^ Moreover, it is important to highlight that the adhesion of cells is facilitated by the proteins present in the serum-containing medium, including albumin, fibronectin, and laminin.^2,53^ Further, these proteins are components of the ECM^2^ and adsorb to the rCFs in a pristine state,^48^ whereby they serve as docking sites for cells via their integrins.^53,54^ The staining of vinculin (see Figure 6b), as a protein that plays a crucial role in cellular adhesion,^55^ allowed the identification of these pronounced cell–cell and cell-fiber contacts when the nonwovens were used in conjunction with Calu-3 cells. Figure 6b further illustrates that the Calu-3 cells display the characteristic morphology of epithelial cells^56^ with close cell contacts as they proliferate along individual fibers. Additionally, employing Calu-3 cells as a relevant *in vitro* model for bronchial epithelium^57,58^ allows their morphology to be interpreted as a promising indicator for tissue development on the nonwoven. Moreover, the cells migrated into the nonwovens and were able to adhere to the entire sample after the seeding process due to the relatively large pore sizes (see Table S1). As illustrated in Figure 6a, cells even formed coherent cell clusters that overgrew and spanned the smallest pores and overlapping fibers, indicating the potential for future tissue formation in the networks. In particular, nonwoven materials with high porosity and larger pore sizes tend to form these large 3D aggregates of cells among the fibers.^2^ Nevertheless, cell morphology, migration, and proliferation are modulated by the alignment of the fibers in the nonwoven material.^59,60^ In the past, studies have demonstrated that the spatial organization of cells in a 3D environment is controlled by pore size.^30,61^ Further, it also serves as a gateway for regulating long-term cellular events essential to tissue development and function. In addition, the abundance of large pores permits the exchange of gases, the delivery of nutrients, and the clearance of metabolic waste products during tissue formation.^62^ Moreover, the rCFs display a significantly increased surface area in comparison to PP fibers due to their smaller diameter. This results in enhanced cell attachment and a superior surface area-to-volume ratio, which is essential for emulating the biological environment and its functions.^29^ These attractive biomimetic features of fibrous structures with smaller fibers are also evident in electrospun nonwoven mats.^63^ However, a significant limitation is that the average pore sizes in these structures are too small, which hinders cell infiltration and severely restricts cell migration through the material’s thickness.^64^ Further, the rCFs exhibit a large variation in tensile properties, potentially influencing the cell proliferation rate, as illustrated in Figure S6.^65^

The potential use of nonwovens as biomaterials in implants is supported by promising indications, including cell organization, cell attachment to each other and to the rCFs, and cluster formation. However, in addition to the aforementioned characteristics, the proliferation and viability of the cells on the nonwovens are also of significant interest. As with cell organization, we utilized Calu-3 cells and, additionally, NIH/3T3 fibroblasts on the nonwoven samples A, B, and C. Fibroblasts have a pivotal function in the synthesis of connective tissue—viz. ECM synthesis—and in tissue repair which is the rationale behind their selection as the second cell line for the viability and proliferation experiments in the context of utilizing the nonwovens as a biomaterial in the lungs.^66^ Initially, we observed that nonwovens did not affect cell viability due to toxic byproducts or partial degradation. Indeed, the viabilities of both cell lines in the nonwoven-exposed medium did not differ significantly from those of the control group, indicating that they are suitable for use as biomaterials.^67^ Since the NIH/3T3 fibroblasts were not imaged with regard to cell organization, it cannot be ruled out that they did not adhere to the PP fibers. However, this does not constitute a limitation in the investigation of viability and proliferation. Our results demonstrate that the viability of NIH/3T3 cells after 4 to 8 days ranged from 80% to 90%, while that of Calu-3 cells was between 60% and 80%. Although the viability was significantly lower than in the control group after the respective periods, it remained sufficiently high for cells to organize in the fiber network and proliferate on the nonwovens. The disparity in viability between the cell lines can be attributed to the fact that the epithelial Calu-3 cells exhibit two-dimensional layer-like growth, which is more challenging when cultivated on a 3D porous scaffold. Consequently, their growth is geometrically impeded. Conversely, the mesenchymal NIH/3T3 cells demonstrate a 3D growth pattern, which facilitates their better adaptation to the 3D porous network. It is also noteworthy that the viability remained at a constant level or even increased in some cases during the longer cultivation period of 8 days compared to 4 days. Given the findings of previous studies, it was reasonable to anticipate that carbon-based materials would have no influence on the viability and proliferation of fibroblasts.^62^ Accordingly, our results suggest that all three nonwoven blends are biocompatible with the chosen cell lines.

Proliferation exhibited a similar pattern, with cell division occurring at consistent levels across all nonwoven blends within the experimental groups. In this context, a proliferation of 0% indicates the cell number at the time of seeding, whereas 100% corresponds to the total number of cells in the control group after 8 days. Here, the resulting cell proliferation illustrates the relative change in relation to this chosen normalization. After 4 days, NIH/3T3 cells exhibited values of approximately 0%, while the Calu-3 cells demonstrated values between −150 and −80%. After 8 days, the NIH/3T3 cells exhibited values in the vicinity of 15–25%, while the Calu-3 cells demonstrated values between 0 and 10%. These results were found to be significantly lower in comparison to the control group. However, in both cell lines, we observed proliferation to increase notably from 4 to 8 days, indicating that the cells were adapting to the environment. One potential explanation for the initial poor proliferation after 4 days is that, due to the large pores, the cells were unable to find a suitable location to adhere or were excluded from the proliferation measurement as a result of falling to the bottom of the well during seeding. Consequently, only a limited number of cells adhered to the nonwovens. This resulted in lower total cell numbers but still high viability values, as the cells remained viable due to the favorable biocompatibility of the nonwovens. The consistent proliferation values per cell line and period for all nonwoven blends may be attributed to the large specific surface area of the rCFs, offering numerous adhesion sites for the cells. This appears to mitigate the influence of the rCF-to-PP fiber ratio on proliferation over 4 and 8 days, thus exhibiting comparable proliferation rates in all the samples. Therefore, it can be concluded that PP fibers do not negatively impact cell viability or proliferation, provided that there is an adequate amount of rCFs and surface area for adhesion or proliferation. Accordingly, the use of different rCF-to-PP fiber ratios may serve as a viable alternative to pure rCF nonwovens, while ensuring that cell viability and proliferation remain unimpaired. However, when used for tissue development, it is crucial to address potential challenges such as insufficient spacing between the rCFs contacts or a limited number of adhesion sites, especially in cases of excessive cell growth. The generally lower viability and proliferation values observed for Calu-3 cells relative to NIH/3T3 cells may be attributed to the Calu-3 cells’ slow growth rate—a typical feature of this cell line^68^—and susceptibility to external factors even in cell culture. Therefore, they may require an extended period to adapt to the nonwoven network environment. In general, the high viability and proliferation of Calu-3 cells and NIH/3T3 fibroblasts across rCF-based nonwovens and their blends with PP fibers demonstrate that both cell lines adhere to the nonwovens, indicating that cell lines with different functions are effectively supported by the fiber network. These findings indicate that the nonwovens could be a suitable biomaterial for tissue development.

## 5 Conclusions

This research has successfully engineered rCF-based needlepunched nonwoven scaffolds and their blends with PP fibers, optimized for interactions with human lung epithelial cells (Calu-3) and murine fibroblast cells (NIH/3T3). These nonwoven scaffolds exhibit tunable porous characteristics, a broad spectrum of fiber orientation distributions, and distinct wetting behavior. Our study demonstrated that bronchial epithelial Calu-3 cells adhere to rCFs, resulting in the development of an epithelial morphology consistent with our findings on laminin adsorption and the wettability of nonwoven materials at varying rCF-to-PP fiber ratios. Although rCFs and PP fibers showed no evidence of cytotoxicity, the limited adhesion potential of PP fibers may cause them to act as insurmountable barriers within the spatial structure of the nonwoven when incorporated in higher proportion. Notably, Calu-3 epithelial cells possessed high viability and proliferation on rCF-based nonwovens, in line with NIH/3T3 cells, making them suitable for artificial lung scaffolds. This exploratory investigation into rCF-based nonwoven scaffolds paves the way for discovering novel applications that address the complex issue of traditional disposal methods, including landfilling and incineration of carbon fiber offcuts, prepreg residues, and semifinished products, thus promoting sustainability. Nonwoven materials with adaptable structures and mechanical properties derived from auxetic characteristics^23^ would further enhance the delivery of oxygen and nutrients, thereby promoting cell proliferation and championing sustainability. In the future, such developments will target multicellular systems with biomimetic precision in their architecture and multiple biological functions, specifically for lung tissue engineering.^69^

## Supporting Information Available

Additional physical properties of the nonwoven samples A, B, and C; additional experimental and statistical results and details; additional fluorescence microscopy images of the nonwoven samples A, B, and C with Calu-3 cells or fibronectin (PDF)
Wetting Behavior: Video of the wetting behavior of the nonwoven samples A, B, and C (AVI)

## Supplementary Material

mt4c01475_si_001.pdf

mt4c01475_si_002.avi
