# Supplementary material for: Sustainable Nonwoven Scaffolds Engineered with Recycled Carbon Fiber for Enhanced Biocompatibility and Cell Interaction: From Waste to Health
Source: ACS Appl Bio Mater. 2025 Feb 17;8(3):1984–96. doi: 10.1021/acsabm.4c01475 (PMC11921018; doi:10.1021/acsabm.4c01475)
Supplement: Supplementary file 1 — mt4c01475_si_001.pdf [file mt4c01475_si_001.pdf]

Supporting Information: "Sustainable Nonwoven Scaffolds Engineered with Recycled Carbon Fiber for Enhanced Biocompatibility and Cell Interaction: From Waste to Health"

Jonas Naumann[1†], Kresten Singer[1†], Siddharth Shukla[2], Alok Maurya[2], Stefan Schlichter[3], Imre Szenti[4], Akos Kukovecz[4], Amit Rawal[2*‡], Mareike Zink[1*‡]

1 Research Group Biotechnology and Biomedicine, Peter-Debye-Institute for Soft Matter Physics, Leipzig University, Linnéstraße 5, 04103 Leipzig, Germany

2 Department of Textile and Fibre Engineering, Indian Institute of Technology Delhi, Hauz Khas, New Delhi, 110016, India

3 Faculty of Mechanical and Process Engineering, Makers labs Recycling & AI, Technische Hochschule Augsburg, University of Applied Sciences, An der Hochschule 1, 86161 Augsburg, Germany

4 Interdisciplinary Excellence Centre, Department of Applied and Environmental Chemistry, University of Szeged, Rerrich Béla tér 1., 6720 Szeged, Hungary

* Corresponding author
Mareike Zink zink@physik.uni-leipzig.de
Amit Rawal arawal@iitd.ac.in

† These authors share first authorship

‡ These authors share last authorship

Materials and Methods

Statistics

The following modules were used for data analysis with python: seaborn, numpy, matplotlib, pandas, and pingouin. [S1-S6] Microsoft Excel was used as a database. [S7] Adobe Illustrator and Adobe Photoshop were used to create and revise the figures. [S8,S9]

Supporting Tables and Figures

Table S1. Physical properties of nonwoven samples.

Sample ID		A	B	C	Source
Composition	rCFs (%)	100	48	6	X-ray microCT
	PP (%)	-	52	94	X-ray microCT
Fibre diameter	rCFs (µm)	7.4 ± 1.1	6.2 ± 1.1	7.0 ± 1.1	SEM
	PP (µm)	-	19.9 ± 2.1	29.6 ± 2.4	SEM
Porosity (%)		96.75	91.4	89.02	X-ray microCT
Mass per unit area (g/m^2)		90	190	325	Measured
Thickness (mm)		1.37±0.23	2.73±0.34	3.76±0.42	Measured
f_p		0.1794	0.0392	0.0334	Calculated
g_p		0.1501	0.0384	0.0329	Calculated
Surface area per unit volume of nonwoven (cm^2/cm^3)		140	200	180	X-ray microCT
Specific surface area of fibre[1] (m^2/g)		0.317	0.262	0.163	Calculated
Mean pore size (µm)		151.6	125.2	113.8	X-ray microCT
Median pore size (µm)		136.4	120.4	109.2	X-ray microCT
Maximum pore size (µm)		371.8	229.6	229	X-ray microCT

Table S2. Parameters of X-ray microCT scans.

Sample ID	Specimen size (mm^2)	Source voltage (kV)	Source current (µA)	Total rotation (º)	Angular step (º)	Exposure time (ms)	Resolution (µm)
A	2 x 2	600	50	194	0.1	250	1.1
B	2.5 x 2.5	600	50	194	0.1	400	1.4
C	2.5 x 2.5	600	50	194	0.1	500	1.4

[1] Specific surface area (SSA) in m^2/g of individual fibres has been calculated using the following equation

$$SSA = \frac{4}{\rho d}$$

where ρ is the density of fibre (g/cm^3) and d is fibre diameter (µm). The density of rCF has been taken as 1.8 g/ cm^3 and the density of PP has been taken as 0.91 g/cm^3 [S10,S11]. The density of fibers has been calculated as the weighted average of the constituent fibers in the case of samples B and C.

Table S3. Experimental groups and the corresponding measured wells.

sample	Time period [days]	Measured wells (viability)		Measured wells (proliferation)	
		Calu-3	NIH/3T3	Calu-3	NIH/3T3
None (control)	0	/	/	6	6
None (control)	4	6	6	6	6
None (control)	8	6	6	6	6
None (nonwoven exposed medium: sample A)	4	6	6	/	/
None (nonwoven exposed medium: sample B)	4	6	6	/	/
None (nonwoven exposed medium: sample B)	4	6	6	/	/
Sample A	4	6	6	6	6
Sample B	4	6	6	6	6
Sample C	4	6	6	6	6
Sample A	8	6	6	6	6
Sample B	8	6	6	6	6
Sample C	8	6	6	6	6

Table S4. P-values of the satistical significance analysis of the viability experiments.

Comparison	Group A	Group B	NIH/3T3	Calu-3
Control 4 days and nonwoven exposed medium 4 days	Exposed; 4 days; A	Exposed; 4 days; C	0.831066	0.246753
	Exposed; 4 days; A	Exposed; 4 days; B	0.198509	1
	Exposed; 4 days; A	Control; 4 days	0.998604	0.720779
	Exposed; 4 days; C	Exposed; 4 days; B	0.155531	0.246753
	Exposed; 4 days; C	Control; 4 days	0.788422	0.246753
	Exposed; 4 days; B	Control; 4 days	0.211463	0.761905
Control 4 days and nonwovens 4 days	Control; 4 days	Nonwoven; 4 days; A	0.002165	0.005462
	Control; 4 days	Nonwoven; 4 days; C	0.002165	0.022543
	Control; 4 days	Nonwoven; 4 days; B	0.002165	0.000344
	Nonwoven; 4 days; A	Nonwoven; 4 days; C	0.041126	0.353822
	Nonwoven; 4 days; A	Nonwoven; 4 days; B	0.484848	0.265058
	Nonwoven; 4 days; C	Nonwoven; 4 days; B	0.025974	0.999782
Control 8 days and nonwovens 8 days	Control; 8 days	Nonwoven; 8 days, A	0.00013	0.000006
	Control; 8 days	Nonwoven; 8 days, C	0.035111	0.002573
	Control; 8 days	Nonwoven; 8 days, B	0.001359	0.001766
	Nonwoven; 8 days, A	Nonwoven; 8 days, C	0.943852	0.054075
	Nonwoven; 8 days, A	Nonwoven; 8 days, B	0.939639	0.062844
	Nonwoven; 8 days, C	Nonwoven; 8 days, B	0.996583	0.955862
Control 4 days and control 8 days	Control; 4 days	Control; 8 days	0.894642	0.077092
Nonwovens 4 days and nonwovens 8 days	Nonwoven; 4 days; A	Nonwoven; 8 days; A	0.453307	0.044772
	Nonwoven; 4 days; B	Nonwoven; 8 days; B	0.625977	0.024411
	Nonwoven; 4 days; C	Nonwoven; 8 days; C	0.393939	0.030372

Table S5. P-values of the satistical significance analysis of the proliferation experiments.

Comparison	Group A	Group B	NIH/3T3	Calu-3
Control 4 days and nonwovens 4 days	Nonwoven; 4 days; C	Nonwoven; 4 days; A	0.0044	0.000223
	Nonwoven; 4 days; C	Nonwoven; 4 days; B	0.133154	0.00015
	Nonwoven; 4 days; C	Control; 4 days	0.000286	1.58E-13
	Nonwoven; 4 days; A	Nonwoven; 4 days; B	0.047361	0.998
	Nonwoven; 4 days; A	Control; 4 days	0.000181	6.06E-11
	Nonwoven; 4 days; B	Control; 4 days	0.000277	7.65E-11
Control 8 days and nonwovens 8 days	Nonwoven; 8 days; C	Nonwoven; 8 days; A	0.768	0.965816
	Nonwoven; 8 days; C	Nonwoven; 8 days; B	0.679	0.242953
	Nonwoven; 8 days; C	Control; 8 days	4.72E-10	0.005004
	Nonwoven; 8 days; A	Nonwoven; 8 days; B	0.999	0.250797
	Nonwoven; 8 days; A	Control; 8 days	1.22E-10	0.005856
	Nonwoven; 8 days; B	Control; 8 days	9.90E-11	0.007663
Control 0 days, control 4 days and control 8 days	Control; 0 days	Control; 4 days	0.00019	0.000206
	Control; 0 days	Control; 8 days	0.000021	0.002946
	Control; 4 days	Control; 8 days	0.196021	0.421497
Nonwovens 4 days and nonwovens 8 days	Nonwoven; 4 days; A	Nonwoven; 8 days; A	0.000015	0.000813
	Nonwoven; 4 days; B	Nonwoven; 8 days; B	0.000174	7.10E-09
	Nonwoven; 4 days; C	Nonwoven; 8 days; C	0.01951	1.47E-09

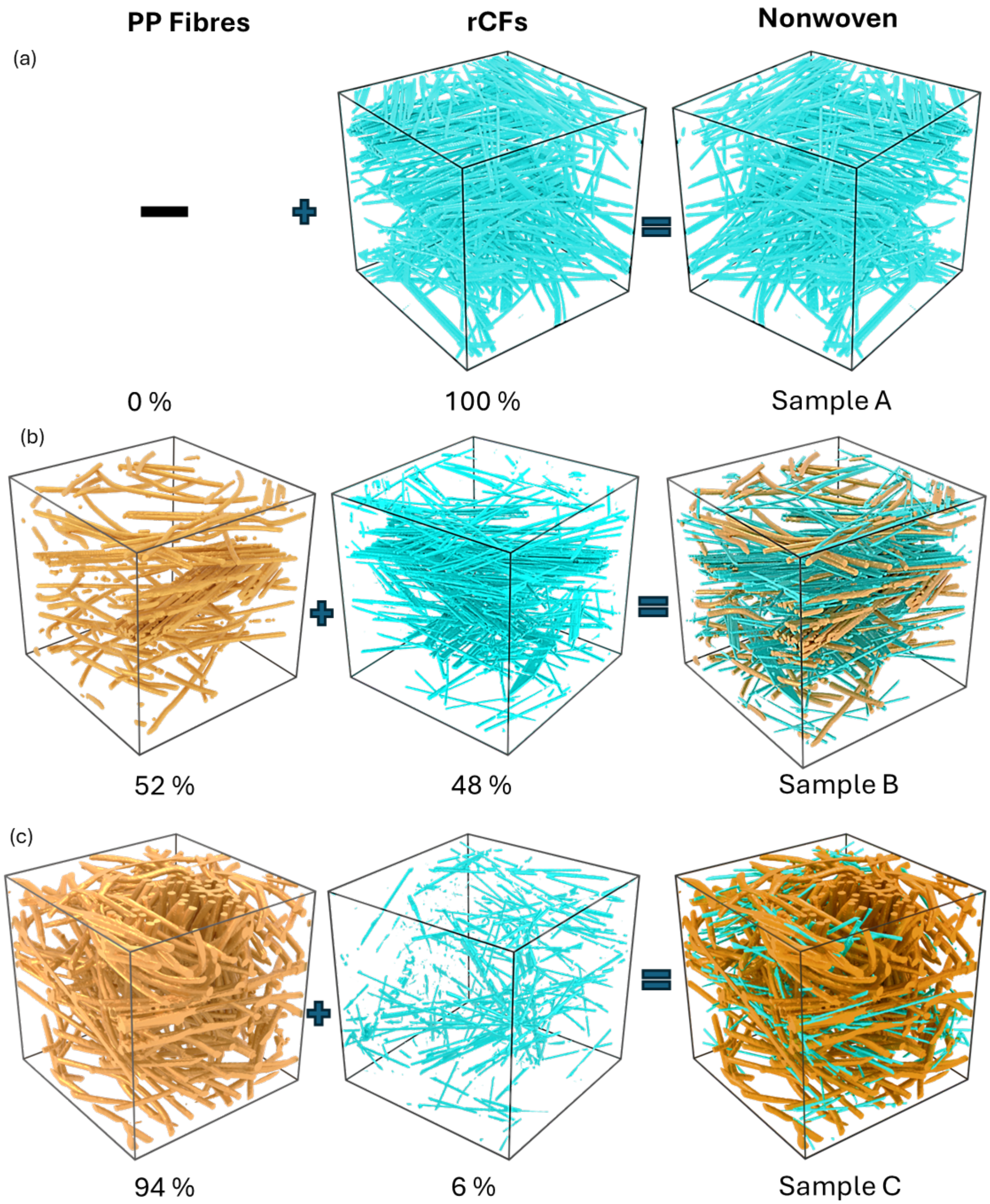

Figure S1. 3D-rendered X-ray microCT images revealing constituent rCFs and PP fibres in the sample **(a)** A, **(b)** B, and **(c)** C.

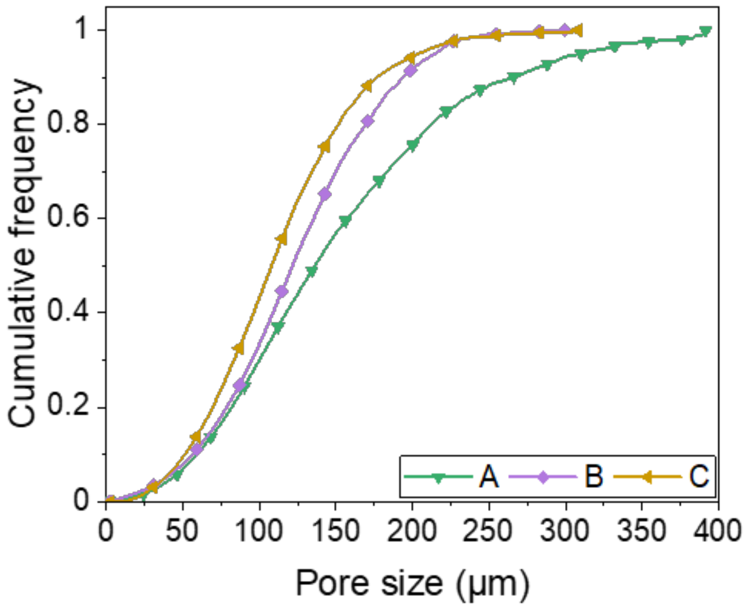

Figure S2. Pore size distribution of samples A, B and C.

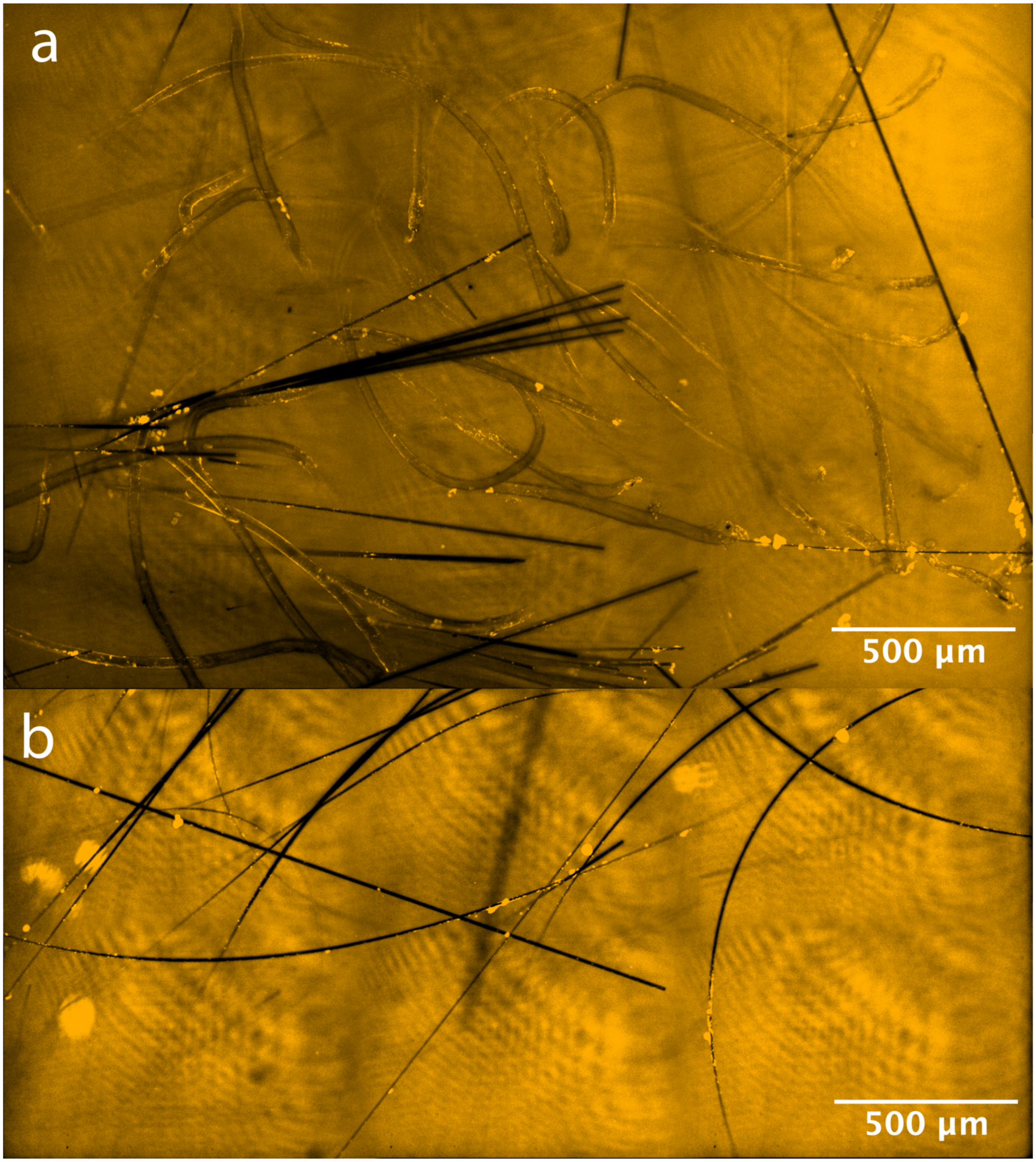

Figure S3. Laminin adsorption on the nonwoven sample **(a)** C and **(b)** A.

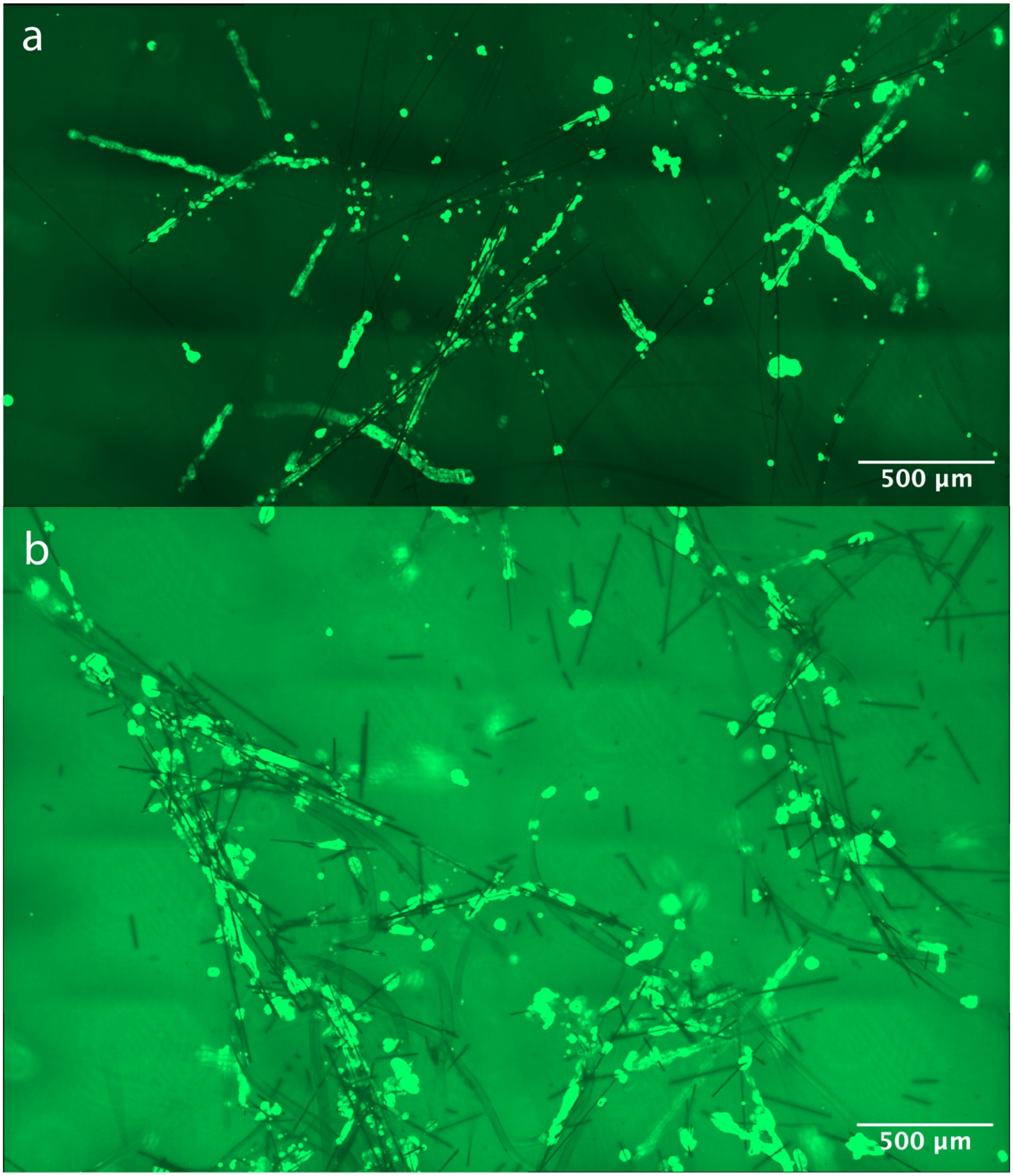

Figure S4. Cell adhesion of Calu-3 cells on the nonwoven sample **(a)** B and **(b)** C.

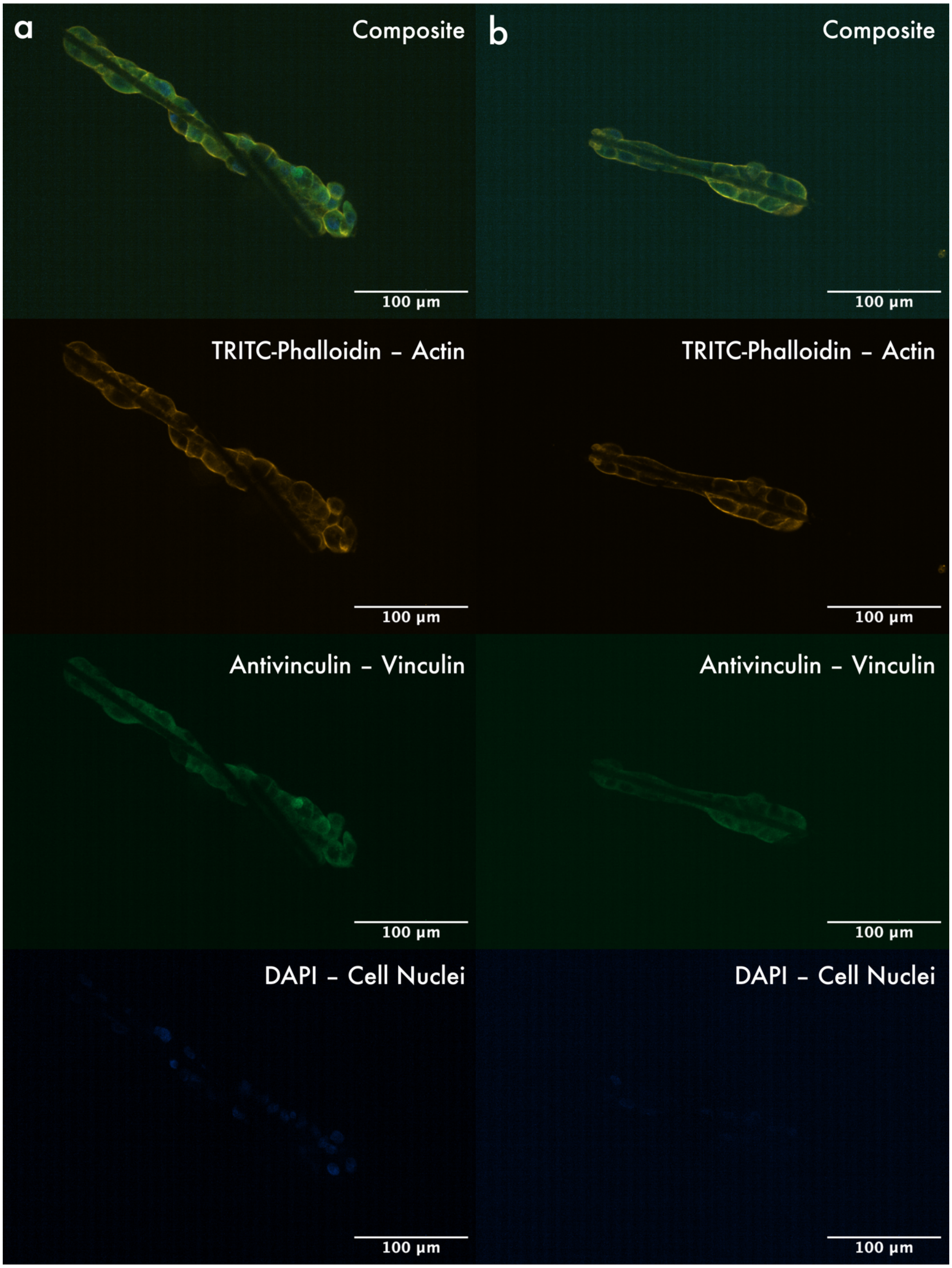

Figure S5. Cell adhesion of Calu-3 cells on rCFs in nonwoven sample **(a)** B and **(b)** C.

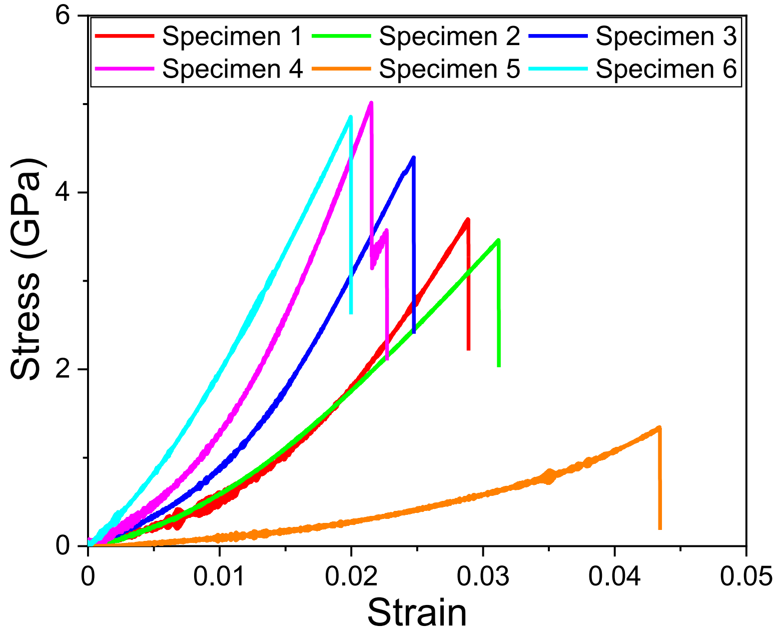

Figure S6. Stress-strain curves of rCFs.

References

[S1] Van Rossum, G.; Drake, F. L. *Python 3 Reference Manual*; CreateSpace: Scotts Valley, CA, **2009**.

[S2] Waskom, M. Seaborn: Statistical Data Visualization. *J. Open Source Softw.* **2021**, *6* (60), 3021. https://doi.org/10.21105/joss.03021.

[S3] The Matplotlib Development Team. Matplotlib: Visualization with Python, **2024**. https://doi.org/10.5281/ZENODO.11201097.

[S4] Harris, C. R.; Millman, K. J.; van der Walt, S. J.; Gommers, R.; Virtanen, P.; Cournapeau, D.; Wieser, E.; Taylor, J.; Berg, S.; Smith, N. J.; Kern, R.; Picus, M.; Hoyer, S.; van Kerkwijk, M. H.; Brett, M.; Haldane, A.; del Río, J. F.; Wiebe, M.; Peterson, P.; Gérard-Marchant, P.; Sheppard, K.; Reddy, T.; Weckesser, W.; Abbasi, H.; Gohlke, C.; Oliphant, T. E. Array Programming with NumPy. *Nature* **2020**, *585* (7825), 357–362. https://doi.org/10.1038/s41586-020-2649-2.

[S5] The pandas development team. Pandas-Dev/Pandas: Pandas, **2024**. https://doi.org/10.5281/ZENODO.10957263.

[S6] Vallat, R. Pingouin: Statistics in Python. *J. Open Source Softw.* **2018**, *3* (31), 1026. https://doi.org/10.21105/joss.01026.

[S7] Microsoft Corporation. Microsoft Excel, **2024**. https://office.microsoft.com/excel.

[S8] Adobe Inc. Adobe Illustrator, **2024**. https://adobe.com/products/illustrator.

[S9] Adobe Inc. Adobe Photoshop, **2024**. https://www.adobe.com/products/photoshop.html.

[S10] Sharma, S.;Rawal, A.; Tóth, I.Y.; Vásárhelyi, L.; Kozma, G.; Kukovecz, Á.; Jee, S.; Ayaydin, F. Superhydrophobic self-similar nonwoven-titanate nanostructured materials. *J. Colloid Interface Sci.* **2021**, 598, 93–103. https://doi.org/10.1016/j.jcis.2021.03.045.

[S11] McNally, T.; Boyd, P.; McClory, C.; Bien, D.; Moore, I.; Millar, B.; Davidson, J.; Carroll, T. Recycled carbon fiber filled polyethylene composites. *J. Appl. Polym. Sci.* **2008**, 107, 2015–2021. https://doi.org/10.1002/app.27253.
